# Integrative lipidomics profile uncovers the mechanisms underlying high-level α-linolenic acid accumulation in *Paeonia rockii* seeds

**DOI:** 10.1093/hr/uhad106

**Published:** 2023-05-15

**Authors:** Weizong Yang, Ziwei Xin, Lihang Xie, Yuhui Zhai, Yanlong Zhang, Lixin Niu, Qingyu Zhang

**Affiliations:** College of Landscape Architecture and Arts, Northwest A&F University, Yangling 712100, Shaanxi, China; College of Landscape Architecture and Arts, Northwest A&F University, Yangling 712100, Shaanxi, China; Academy of Medical Sciences, Zhengzhou University, Zhengzhou 450000, China; College of Landscape Architecture and Arts, Northwest A&F University, Yangling 712100, Shaanxi, China; College of Landscape Architecture and Arts, Northwest A&F University, Yangling 712100, Shaanxi, China; College of Landscape Architecture and Arts, Northwest A&F University, Yangling 712100, Shaanxi, China; College of Landscape Architecture and Arts, Northwest A&F University, Yangling 712100, Shaanxi, China

## Abstract

Tree peony (*Paeonia rockii*) is an excellent woody oilseed crop, known for its high α-linolenic acid (ALA, ~45%) content, which is of great value for human health. However, the mechanisms underlying this high-level ALA accumulation in tree peony seeds are poorly understood. In this study, we evaluated the dynamic changes in the lipidomic profile of *P. rockii* seeds during development. A total of 760 lipid molecules were identified in *P. rockii* seeds; triacylglycerol (TAG) lipid molecules showed the highest abundance and diversity, both increasing during seed development. Particularly, ALA was the predominant fatty acid at the TAG *sn*-3 position. We further characterized two diacylglycerol acyltransferase (DGAT) genes and three phospholipid:diacylglycerol acyltransferase (PDAT) genes involved in the transfer of fatty acids to the TAG *sn*-3 position. Gene expression and subcellular localization analyses suggested that PrDGATs and PrPDATs may function as endoplasmic reticulum-localized proteins in seed TAG biosynthesis. *In vitro* functional complementation analysis showed different substrate specificities, with PrPDAT2 having a specific preference for ALA. Multiple biological assays demonstrated that PrDGAT1, PrDGAT2, PrPDAT1-2, and PrPDAT2 promote oil synthesis. Specifically, PrPDAT2 leads to preferential ALA in the oil. Our findings provide novel functional evidence of the roles of *PrDGAT1* and *PrPDAT2*, which are potential targets for increasing the ALA yield in tree peony and other oilseed crops.

## Introduction

Tree peony is a renowned ornamental and medicinal plant widely cultivated in China as a novel woody oilseed crop [1, 2]. The cultivation area of oil tree peony exceeds 129 333 hectares, resulting in an annual seed oil production of 53 000 tons. Tree peony seeds are a nutritious source of edible oils, being especially rich in α-linolenic acid (ALA), which accounts for ~45% of total fatty acid (FA) content [3, 4]. Importantly, ALA is essential for human nutrition and health, and it can only be synthesized by plants [5]. For these reasons, tree peony is a potentially valuable oilseed crop worldwide.

In nature, most plants have low ALA levels; thus, increasing the ALA content is necessary to improve the quality of oilseed crops. Previous studies on ALA accumulation mainly focused on model plants and the specific genes responsible for high ALA germplasm are not well characterized [6, 7]. As one of the few high-ALA woody oilseed crops, tree peony may be a particular model for ALA accumulation. Therefore, investigating the mechanisms underlying the high-level ALA accumulation in tree peony seeds has considerable foundational and strategic importance.

In higher plants, ALA accumulation in seeds mainly involves three metabolic pathways, namely acyl editing, the Kennedy pathway, and the acyl-CoA-independent pathway [8]. In the first, ω-3 FA desaturase 3 (FAD3) catalyzes linoleic acid (LA) to synthesize ALA. Most of the synthesized ALA requires assembly to the glycerol backbone of triacylglycerol (TAG), which involves the other two main pathways. *In planta*, diacylglycerol acyltransferase (DGAT) and phospholipid:diacylglycerol acyltransferase (PDAT) are the only enzymes responsible for FA assembly at the *sn*-3 position of TAG [9]. In addition, the selectivity of DGAT and PDAT for different acyl groups causes changes in the FA composition [10]. In some germplasms rich in polyunsaturated fatty acids (PUFA), PDAT effectively transfers highly desaturated FAs from phosphatidylcholine (PC) to TAG [10]. In *Arabidopsis dgat1* mutant seeds, higher *AtPDAT1* expression increased the ALA content by 1-fold [11]. In *Camelina sativa* seeds, the lower *DGAT1* expression due to amiRNA (microRNA) increased ALA content, while *PDAT* overexpression significantly decreased ALA content [12]. Overexpression of flax *LuDGAT2* in yeast increased ALA content by 50% [13] whereas *Cyperus esculentus CeDGAT2* expression reduced the ALA content in tobacco leaves [14]. Thus, DGAT and PDAT possess distinct substrate specificities in different plants, determining their role in ALA accumulation.

In this study, the analysis of dynamic changes in lipids during *Paeonia rockii* seed development using ultra-performance liquid chromatography–tandem mass spectrometry (UPLC–MS/MS) showed the highest abundance and diversity of TAGs, which increased with seed development. Moreover, ALA is mainly incorporated into the *sn-*3 position of TAG. Further research on the mechanisms of the high-level ALA accumulation in *P. rockii* seeds revealed the involvement of a novel major gene, *PrPDAT2*, in ALA accumulation. An *in vitro* functional complementation assay showed PrPDAT2’s specific transfer preference for ALA. *PrPDAT2* overexpression promoted ALA accumulation in both transgenic *Nicotiana benthamiana* leaves and stable transgenic *Arabidopsis thaliana* seeds, as further confirmed by gene silencing in *P. rockii* leaves. In addition, PrDGAT1 was also involved in ALA accumulation in seed oil. Together, our results provide new insights into the potential mechanisms underlying high-level ALA accumulation in tree peony seeds, highlighting the role of PrPDAT2.

## Results and discussion

### Lipidomics profile of *P. rockii* seeds

To investigate the mechanisms underlying the high ALA accumulation, we analyzed the lipidomic profile of *P. rockii* seeds during development using UPLC–MS/MS. The quality of the acquired data was assessed by quality control (QC) repeatability, as shown in Supplementary Data Fig. S1. A total of 760 lipid molecules were identified from *P. rockii* seeds (Table 1, Supplementary Data Table S1) and categorized into six lipid classes, including 398 glycerolipids (GLs), 214 glycerophospholipids (GPs), 58 saccharolipids (SLs), 63 sphingolipids (SPs), 3 prenol lipids (PLs), and 24 fatty acyls (Table 1, Supplementary Data TableS1). In comparison, the lipid molecules of hickory had 98 GLs, 383 GPs, 51 SLs, 10 SPs, and 2 fatty acyls, whereas peanut contained 156 GLs, 112 GPs, 93 SPs, and 17 fatty acyls [15, 16]. It was noteworthy that tree peony featured PLs, lacking in both hickory and peanut samples, and the GL lipid molecules of tree peony were more abundant.

The lipid compounds of *P. rockii* were further classified into 27 subclasses, comprising 311 TAGs, 66 diacylglycerols (DAGs), 49 phosphatidylethanolamines (PEs), 30 phosphatidylcholines (PCs), 26 monogalactosyldiacylglycerols (MGDGs), 25 phosphatidylinositols (PIs), and other lipid molecules (Table 1). TAG had the most abundant and diverse variety of lipid molecules among all subclasses. Next, we compared tree peony with other worldwide common oil crops soybean—peanut, sesame, and rapeseed; our samples contained many more TAG molecules than any of them [15]. These data showed the abundant diversity of lipid compositions in *P. rockii* seeds, particularly with respect to TAG lipid molecule species.

**Table 1:** Information on identified lipids in *P. rockii* seeds.

Category	Main class	Subclass	Number
Glycerolipids	Neutral glycerolipid	Triacylglycerol	311
Glycerolipids	Neutral glycerolipid	Diacylglycerol	66
Glycerolipids	Neutral glycerolipid	Monoglyceride	4
Glycerolipids	Neutral glycerolipid	Diacylglycerol trimethylhomoserine	15
Glycerolipids	Neutral glycerolipid	Diacylglycerol-3-*O*-carboxymethyl choline	1
Glycerolipids	Neutral glycerolipid	Diacylglycerol glucuronic acid	1
Glycerophospholipids	P-Ethanol amine	Phosphatidylethanolamine	49
Glycerophospholipids	P-Ethanol amine	Lysophosphatidylethanolamine	7
Glycerophospholipids	P-Choline	Phosphatidylcholine	30
Glycerophospholipids	P-Choline	Lysophosphatidylcholine	18
Glycerophospholipids	P-Acid	Phosphatidic acid	16
Glycerophospholipids	P-Acid	Lysophosphatidic acid	7
Glycerophospholipids	P-Methanol	Phosphatidyl carbinol	9
Glycerophospholipids	P-Inositol	Phosphatidylinositol	25
Glycerophospholipids	P-Inositol	Lysophosphatidylglycerol	8
Glycerophospholipids	P-Serine	Phosphatidylserine	13
Glycerophospholipids	P-Glycerol	Phosphatidylglycerol	27
Glycerophospholipids	P-Glycerol	Lysophosphatidylglycerol	5
Saccharolipids	Glycoglycerolipid	Monogalactosyldiacylglycerol	26
Saccharolipids	Glycoglycerolipid	Digalactosyldiacylglycerol	17
Saccharolipids	Glycoglycerolipid	Sulfoquinovosyldiacylglycerol	15
Sphingolipids	Glycosphingolipids	Phytoceramides	24
Sphingolipids	Glycosphingolipids	Ceramides	19
Sphingolipids	Neutral glycosphingolipids	Hexosylceramides, HexCer	16
Sphingolipids	Neutral glycosphingolipids	Sphingosine	4
Prenol lipids	Quinones and hydroquinones	Coenzyme Q	3
Fatty acyls	Fatty acid	Free fatty acid	24

### Lipidomic changes in *P. rockii* seeds during seed development

**Figure 1:**
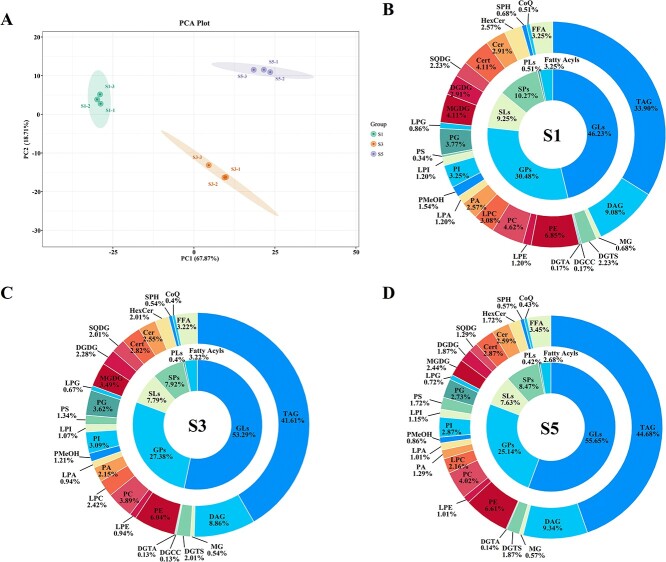
Changes in number of lipid molecules identified in *P. rockii* seeds during development. (A) PLS-DA score plot comparing *P. rockii* seeds during development. (B–D) Number of lipid molecules identified in *P. rockii* seeds at the S1 (B), S2 (C), and S3 (D) stages.

We further investigated the differences in lipidomic profiles at three developmental stages of *P. rockii* seeds using partial least squares discriminant analysis (PLS-DA). The PLS-DA scores plot revealed an obvious separation between the lipid profiles of different developmental stages in tree peony seeds (Fig. 1A). Major drivers for this separation were the first two principal components, DAG and TAG, suggesting marked differences in DAG and TAG content in seeds at different developmental stages.

Next, we analyzed the dynamic changes of lipid molecule species. During seed development, the percentage of GLs gradually increased, while SPs slightly decreased and then increased, and the percentages of GPs, SLs, and fatty acyl species decreased, with unaltered low levels of PLs. More specifically, the proportions of GLs, GPs, SPs, SLs, fatty acyls, and PLs were 46.23, 30.48, 10.27, 9.25, 3.25, and 0.51%, respectively, during the S1 period (Fig. 1B) and 53.29, 27.38, 7.92, 7.79, 3.22, and 0.4%, respectively, in the S3 period (Fig. 1C); by the S5 period they were 55.65, 25.14, 8.47, 7.63, 2.68, and 0.42%, respectively (Fig. 1D). Notably, the variety of TAG lipid molecules was relatively greater with seed development at the subclass level. This may be attributed to an in-depth desaturation of FAs during seed development, consistent with our previous finding that major unsaturated fatty acids (UFAs) accumulate with seed maturation in tree peony seeds [17].

**Figure 2:**
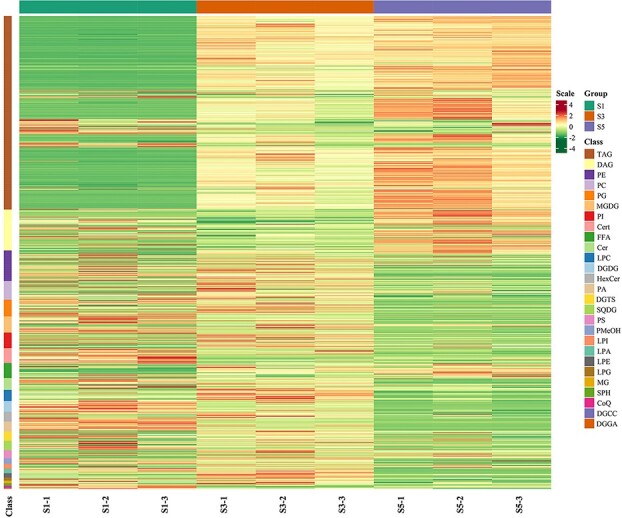
Heat map of dynamic changes in lipid contents of *P. rockii* seeds during development. Lipid molecule contents in the heat map are normalized by row, and red and green represent increase and decrease of lipid molecule content, respectively.

The dynamic changes in lipid content of *P. rockii* seeds at different developmental stages are shown in Fig. 2. Notably, there was a significant increase in TAGs and DAGs during seed development. Conversely, the content of digalactosyldiacylglycerols (DGDGs), hexosylceramides (HexCers), ceramides (Cers), phytoceramides (Certs), diacylglycerol-3-*O*-carboxymethyl cholines (DGCCs), diacylglycerol glucuronic acids (DGGAs), lysophosphatidic acids (LPAs), phosphatidic acids (PAs), PEs, phosphatidylglycerol (PGs), phosphatidyl carbinols (PMeOHs), and sulfoquinovosyldiacylglycerols (SQDGs) decreased gradually from S1 to the mature stage. The content of DGTs, lysophosphatidylcholines (LPCs), lysophosphatidylethanolamines (LPEs), PCs, PIs, and phosphatidylserines (PSs) initially increased during seed development but decreased when the seeds were fully mature. The remaining components, such as coenzyme Q (CoQ), sphingosines (SPHs), lysophosphatidylglycerols (LPGs), lysophosphatidylglycerols (LPIs), monoglycerides (MGs), and free fatty acids (FFAs), showed little changes in content during seed maturation and remained almost constant. As the PC *sn-*2 position is the site for FA desaturation, their dynamic changes contribute to the production of PUFAs such as ALA [9]. As the seeds mature, an increasing amount of PA catalyzed by PA phosphatase generates DAG to provide a substrate for TAG synthesis, which may explain the decreasing PA content. A similar trend was found in *Carya cathayensis* and *Buglossoides arvensis* seed development [16, 18].

### Fatty acid localization at *sn*-1, *sn*-2, and *sn*-3 of triacylglycerol molecules

**Figure 3:**
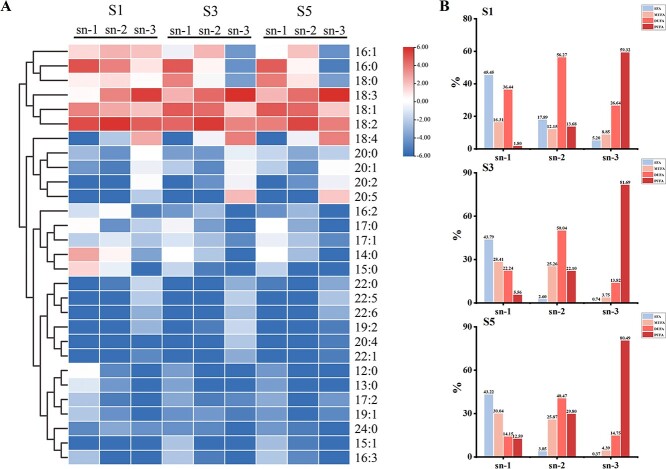
. Localization of FAs at *sn*-1, *sn*-2, and *sn*-3 positions in TAG lipid molecules of *P. rockii* seeds during development. (A) Localization of FAs on TAG lipid molecules. (B) Percentage of SFA, MUFA, DUFA, and PUFA in the different positions of TAG.

Among the lipid components of *P. rockii* seeds, TAG was the most abundant and diverse class, which is consistent with the findings in the worldwide common oil crops soybean, peanut, sesame, and rape [15, 19]. To uncover the biosynthetic mechanisms underlying the high-level ALA accumulation in *P. rockii* seeds, we analyzed the distribution specificity of FAs in TAG. As shown in Fig. 3A, the TAG molecules were primarily composed of C16:0, C18:0, C18:1, C18:2, and C18:3, especially C18:2 and C18:3 UFAs, which is consistent with our previous reports that tree peony seed oil is rich in UFAs [20]. The main FAs at the *sn*-1 position of TAG were C16:0, C18:1, and C18:2, but the proportion of C18:1 gradually increased during seed development. At the *sn*-2 position, there were mainly C16:0, C18:2, and C18:3 during the S1 stage and C18:1, C18:2, and C18:3 in the middle and late stages of seed development. Interestingly, the C18:3 ratio at the *sn*-3 position of TAG was the highest throughout seed development compared with the other FAs (Fig. 3A). However, in the LA-rich hickory, the TAG *sn-*3 position was mainly C18:2 [16], which suggests that the distribution specificity of FAs in TAGs varies among plants and has a vital effect on the FA composition.

Furthermore, we analyzed the percentages of saturated fatty acids (SFA), mono-unsaturated fatty acids (MUFA), di-unsaturated fatty acids (DUFA), and PUFA at the different TAG positions. As shown in Fig. 3B, SFA at the TAG *sn-*1 position showed the highest percentage throughout seed development. It has been reported that SFA at the TAG *sn-*1 position may help reduce cholesterol absorption [21]. On the other hand, the DUFA percentage was highest at the TAG *sn-*2 position, increasing with seed development (Fig. 3B). PUFA was the FA with the most significant percentage at the TAG *sn-*3 position throughout seed development. Especially in the middle and late stages of seed development, the percentage of PUFA was >80% at the TAG *sn-*3 position; in particular, ALA was the FA with the highest PUFA abundance (Fig. 3A and B). Given that acyltransferase is responsible for FA assembly in TAG *in planta* [9], we hypothesize that the acyltransferase responsible for the transfer of FAs to the TAG *sn-*3 position may contribute to ALA accumulation in tree peony seed oil.

### Identification of PrDGATs and PrPDATs in *P. rockii*

**Figure 4:**
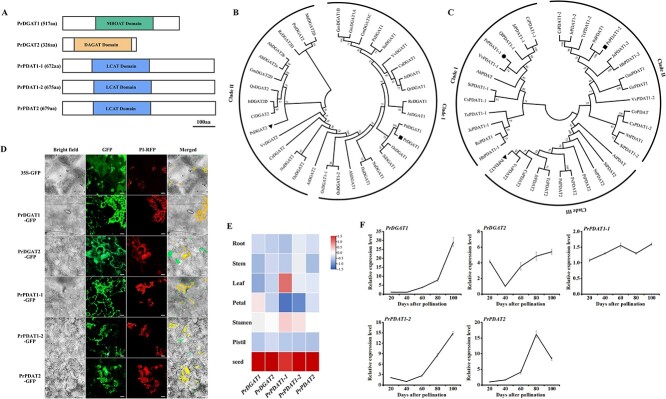
Molecular characterization of PrDGATs and PrPDATs. (A) Schematic representation of the structure of PrDGATs and PrPDATs. aa, amino acids. (B and C) Phylogenetic tree of PrDGATs (B) and PrPDATs (C). Pr, *Paeonia rockii*; Pd, *Paeonia delavayi*; Ca, *Corylus americana*; Ps, *Prunus sibirica*; Jr, *Juglans regia*; Vv, *Vitis vinifera*; So, *Syzygium oleosum*; Oe, *Olea europaea*; Qr, *Quercus robur*; Si, *Sesamum indicum*; Ah, *Arachis hypogaea*; Os, *Oryza sativa*; Ca, *C. americana*; Na, *Nicotiana attenuata*; Ha, *Helianthus annuus*; Gm, *Glycine max*; Rc, *Ricinus communis*; At, *Arabidopsis thaliana*; Cs, *Camellia sinensis*; Qs, *Quercus suber*; Pm, *Prunus mume*; Ci, *Carya illinoinensis*; Md, *Malus domestica*; Rc, *Rosa chinensis*; Hb, *Hevea brasiliensis*; Jc, *Jatropha curcas*; Ql, *Quercus lobata*; Co, *Camellia oleifera*; Tc, *Theobroma cacao*; Gs, *Glycine soja*; Nt, *Nicotiana tabacum*; Pt, *Populus trichocarpa*. (D) Subcellular localization assay of PrDGATs and PrPDATs. Bars = 5 μm. (E) Tissue-specific expression of *PrDGAT*s and *PrPDAT*s. (F) Temporal expression pattern of *PrDGAT*s and *PrPDAT*s in developing seeds.

The final step of TAG synthesis to be completed involved the catalysis of PrDGATs and PrPDATs. Our data showed that ALA is the predominant FA at the TAG *sn*-3 position, which suggests that PrDGATs and PrPDATs may prefer to incorporate ALA into TAG. Thus, we identified the gene sequences of *PrDGAT*s and *PrPDAT*s, including *PrDGAT1*, *PrDGAT2*, *PrPDAT1-1*, *PrPDAT1-2*, and *PrPDAT2*, by BLASTN from our previous transcriptome data of *P. rockii* seeds using *Arabidopsis* homologs as queries [17]. The coding sequences of *PrDGAT1*, *PrDGAT2*, *PrPDAT1-1*, *PrPDAT1-2*, and *PrPDAT2* were 1554, 981, 2019, 2028 and 2040 bp in length, respectively (Supplementary Data Appendix S1). *PrDGAT1* and *PrDGAT2* encode 517- and 326-amino acid proteins, which contain a membrane-bound *O*-acyltransferase (MBOAT) domain and diacylglycerol acyltransferase (DAGAT) domain, respectively (Fig. 4A). *PrPDAT1-1*, *PrPDAT1-2*, and *PrPDAT2* encode proteins containing a lecithin:cholesterol acyltransferase (LCAT) domain and comprise 672, 675, and 679 amino acids, respectively (Fig. 4A).

To gain insights into evolutionary and functional associations, we obtained their homologous protein sequences from the NCBI database via BLASTP and constructed a phylogenetic tree. Evolutionary analysis showed that PrDGAT1 and PrDGAT2 belong to the DGAT1 clade (clade I) and DGAT2 clade (clade II), respectively (Fig. 4B), while PrPDAT1-1, PrPDAT1-2, and PrPDAT2 are clustered into the PDAT1-1 clade (clade I), PDAT1-2 clade (clade II), and PDAT2 clade (clade III), respectively (Fig. 4C). PrDGATs and PrPDATs showed high homology with acyltransferases from *Camellia sinensis*, *Olea europaea*, and *Glycine max*, all typical oilseed crops. These data suggest that the PrDGATs and PrPDATs isolated from tree peony may be involved in oil synthesis.

As shown in Supplementary Data Fig. S2A, PrDGAT1 possesses conserved motifs essential for its acyltransferase function, such as the acyl-CoA binding motif, DAG binding motif, FA protein signature motif, and HXXXXD/N motif. Among them, the HXXXXD/N motif directly affects the acyltransferase activity [22]. The PrDGAT2 alignment showed some highly conserved motifs in DGAT2 of plants, such as the PH motif, PR motif, GGE motif, VPFG motif, and G motif (Supplementary Data Fig. S2B) [23, 24]. All three PrPDATs have Trp residues in the catalytic triad (Ser-Asp-His) and lipase cap structural domains, which directly determine their FA transfer activity [25]. In addition, all PrDGATs and PrPDATs share a highly conserved ER motif, implying that they may localize to the endoplasmic reticulum (ER), the organelle involved in TAG synthesis (Supplementary Data Fig. S2C and D) [9].

### PrDGATs and PrPDATs are all endoplasmic reticulum-localized proteins

*In planta*, TAG synthesis occurs in the ER. To determine the localization of PrDGATs and PrPDATs in cells, they were fused with green fluorescent protein (GFP) and expressed in *N. benthamiana* leaves. As shown in Fig. 4D, the GFP signals from PrDGATs-GFP or PrPDATs-GFP co-localized with the RFP signal from the ER localization protein NtERMP1-RFP, whereas the 35S-GFP positive control was found throughout the cells. These data demonstrate that PrDGATs and PrPDATs are all ER-localized proteins.

Previously, DGAT1 and DGAT2 were isolated from the tung tree, localized to the ER, and both were capable of synthesizing TAG [26]. PDAT1-2 isolated from castor bean localized to the ER and was active in TAG synthesis in transgenic *Arabidopsis* [27]. Recently, the PDAT isolated from *Nannochloropsis oceanica* was shown to be an ER-localized protein, involved in incorporating FAs into TAG [28]. In our study, the subcellular localization assay suggests that PrDGATs and PrPDATs isolated from *P. rockii* may be involved in TAG synthesis.

### *PrDGAT*s and *PrPDAT*s are expressed specifically in seeds

To further understand the biological characteristics of *PrDGAT*s and *PrPDAT*s, we examined their spatial and temporal expression patterns using RT–qPCR. Our data show that *PrDGAT*s and *PrPDAT*s were more strongly expressed in seeds than in other tissues, whereas *PrPDAT1-1* had higher expression levels in leaves (Fig. 4E). Interestingly, in addition to seeds, they were also expressed at relatively high levels in stamens. Previous studies found *DGAT1* and *PDAT1* were highly expressed in *Arabidopsis* pollen, and *DGAT1* and *PDAT1* deletion caused the loss of pollen vitality [29]. Moreover, TAG has been shown to be essential for the reproductive activity of pollen [30]. Therefore, based on our data, we speculate that PrDGATs and PrPDATs may be involved in TAG synthesis, especially in the seed.

As shown in Fig. 4F, the temporal expression patterns of *PrDGAT1* and *PrPDAT1-2* were similar, with low expression levels at early seed development (20–40  days after pollination (DAP)) then increasing until peaking at 100 DAP (Fig. 4F). *PrDGAT2* had a high expression level at 20 DAP, followed by a marked decrease at 40 DAP, then slowly increasing to reach a peak at 100 DAP (Fig. 4F). Notably, *PrPDAT1-1* maintained a steady and low expression level throughout seed development (Fig. 4F). *PrPDAT2* expression was low at early seed development (20–40 DAP), followed by a small increase at 60 DAP, then increased extremely rapidly until it peaked at 80 DAP, and subsequently decreasing slightly at 100 DAP (Fig. 4F). Interestingly, *PrDGAT1*, *PrDGAT2*, *PrPDAT1-2*, and *PrPDAT2* were all expressed at relatively high levels at the middle and late periods of seed development, the stages of high-level seed oil accumulation [17]. Thus, the dynamic expression patterns of *PrDGAT1*, *PrDGAT2*, *PrPDAT1-2*, and *PrPDAT2* suggest their involvement in oil synthesis.

### PrDGATs and PrPDATs have different substrate specificities in *Saccharomyces cerevisiae* H1246

**Figure 5:**
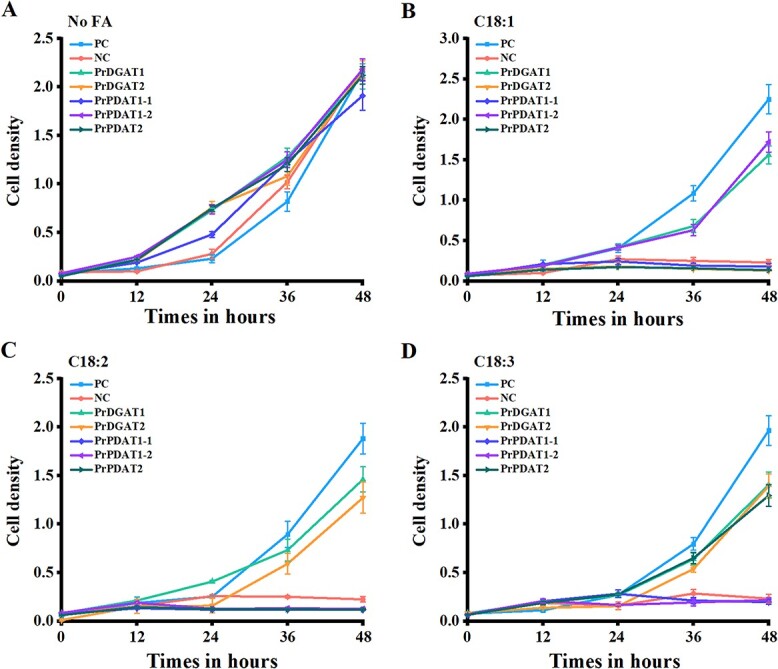
Substrate preference assays of PrDGATs and PrPDATs performed in H1246. (A–D) Growth curve of H1246 cultured in medium supplemented with no free fatty acid (No FA) (A), oleic acid (C18:1) (B), linoleic acid (C18:2) (C), and α-linolenic acid (C18:3) (D). The optical density of the cultures at 600 nm represents cell density.

*Saccharomyces cerevisiae* mutant H1246 cannot synthesize TAG due to the deletion of the acyltransferase gene that controls TAG biosynthesis [31]. H1246 will experience lipotoxicity and not grow when medium is supplemented with free FAs. Accordingly, H1246 is frequently used to investigate the substrate preference of DGAT and PDAT [25, 26]. To determine the transfer preference of PrDGATs and PrPDATs for FAs, we performed a TAG synthesis functional complementation assay in H1246. C18:1, C18:2, and C18:3 were selected for substrate specificity assay as the TAG *sn*-3 position in mature seeds mainly corresponds to UFAs, especially with an extremely high proportion of ALA (Fig. 3B). Our data show that H1246 expressing *PrDGAT*s or *PrPDAT*s can grow normally in media without FA addition, like positive and negative controls (Fig. 5A). When C18:1 was added to the medium, only H1246 expressing *PrDGAT1* or *PrPDAT1-2* and the positive control could rescue the lipotoxicity (Fig. 5B). Only H1246 expressing *PrDGAT1* or *PrDGAT2* showed a healthy growth curve in medium supplemented with C18:2, as did the positive control (Fig. 5C). Moreover, H1246 expressing *PrDGAT1*, *PrDGAT2*, or *PrPDAT2* in a medium supplemented with C18:3, could rescue the lipotoxicity, restoring growth (Fig. 5D). Our data suggest that the PrDGATs and PrPDATs isolated from tree peony have different substrate specificities in yeast mutants. Specifically, PrDGAT1 and PrDGAT2 had a wide range of FA preferences and can both transfer C18:2 and C18:3, but PrDGAT1 can also transfer C18:1. In contrast, PrPDAT1-2 and PrPDAT2 had a unique FA preference for C18:1 and C18:3, respectively. However, PrPDAT1-1 showed no C18:1, C18:2, and C18:3 transferability, consistent with our hypothesis that it may not be involved in TAG synthesis based on the *PrPDAT1-1* expression pattern.

Previous studies have found that some acyltransferases can transfer a wide range of FAs. DGAT1 and DGAT2 isolated from the tung tree can transfer C18:1, C18:2, and C18:3 to synthesize TAG, although DGAT1 showed a higher preference for C18:2 [26]. In *B. arvensis*, DGAT1, DGAT2, PDAT1, and PDAT2 all can transfer FAs to synthesize TAG; particularly DGAT2 showed a more intense ability to incorporate PUFA [18]. However, DGAT2 isolated from *C. esculentus* showed a unique transfer preference for C18:1 [14]. In this study, although PrDGAT1 and PrDGAT2 could transfer C18:3, they also showed a wide range of FA preferences, which may limit their activity in ALA accumulation. Interestingly, PrPDAT2 showed a unique FA transfer preference for C18:3; thus, PrPDAT2 may play a key role in ALA accumulation in tree peony seeds.

### Transient *PrDGAT* and *PrPDAT* overexpression enhance oil content and change fatty acid composition in *N. benthamiana*

**Figure 6:**
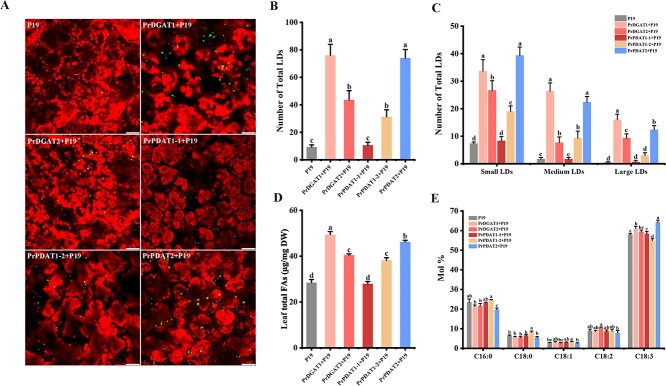
Overexpression of *PrDGAT*s and *PrPDAT*s enhances oil content and changes FA composition in *N. benthamiana* leaves. (A) Images of LDs in *N. benthamiana* leaf tissue. LDs are shown in green and chloroplasts are shown in red. Image area: 232.50 × 232.50 μm^2^. Bars = 40 μm. (B) Number of total LDs per image area in P19 control and *PrDGAT*/*PrPDAT*-overexpressing *N. benthamiana* leaf tissue. (C) Number of LDs of different size classes in each image area. Small LDs, Nile Red-dyed lipid area <3 μm^2^; medium LDs, 3–6 μm^2^; large LDs, 6–10 μm^2^. (D) Total FA contents in P19 control and *PrDGAT*/*PrPDAT*-overexpressing *N. benthamiana* leaves. (E) Fatty acid composition of P19 control and *PrDGAT*/*PrPDAT*-overexpressing *N. benthamiana* leaves. DW, dry weight. Values are mean ± standard deviation (*n* = 3). Different letters indicate significant difference at *P* < .05, as determined by Tukey’s post-test.

A previous study investigated the functions of *Arabidopsis* SEIPIN proteins in oil synthesis using the transient expression system of *N. benthamiana* [32]. To examine the role of PrDGATs and PrPDATs in oil synthesis *in planta*, we performed a similar assay in *N. benthamiana. PrDGAT* and *PrPDAT* transcript accumulation in tobacco leaves was confirmed by RT–PCR at 6 days after infiltration (dpi) (Supplementary Data Fig. S3). As shown in Fig. 6A, except for PrPDAT1-1, PrDGATs and PrPDATs induced accumulation of more lipid droplets (LDs) in the leaves than in P19 control leaves. Further LD quantification showed a higher total LD number in leaves expressing *PrDGAT1* or *PrPDAT2*, ~8.1- and ~ 7.9-fold more than in the P19 control, respectively (Fig. 6B). The total LD number in leaves expressing *PrDGAT2* or *PrPDAT1-2* also increased to ~4.6- and ~ 3.3-fold more than in P19 control, respectively, whereas *PrPDAT1-1* expression did not change the LD number (Fig. 6B). Consistently, PrDGAT1, PrDGAT2, PrPDAT1-2, or PrPDAT2 significantly increased the numbers of small, medium, and large LDs in tobacco leaves compared with the P19 control (Fig. 6C). Furthermore, PrPDAT2 mostly increased the number of small LDs, while PrDGAT1 mostly increased medium and large LDs (5.4-, 15.8-, and 48-fold more than in the P19 control, respectively) (Fig. 6C). These results suggest that, except for PrPDAT1-1, PrDGATs and PrPDATs induce LD accumulation.

Previous studies have found that increased LD numbers contribute to oil accumulation [32]. Consistent with the LD accumulation observed in leaves overexpressing *PrDGAT*s and *PrPDAT*s, their oil levels were significantly increased. Compared with the P19 control, PrDGAT1 and PrPDAT2 increased the oil content to a greater extent (73 and 62%, respectively) than in PrDGAT2 and PrPDAT1-2 (42 and 34%, respectively) (Fig. 6D, Supplementary Data Table S2). These data suggest that PrDGAT1, PrDGAT2, PrPDAT1-2, and PrPDAT2 were involved in oil synthesis. Particularly, PrDGAT1 and PrPDAT2 had higher involvement than other proteins.

Considering the different FA preferences of PrDGATs and PrPDATs obtained in the yeast assay, we further analyzed the FA composition of transgenic leaves. Oil accumulation was accompanied by changes in FA composition in leaves overexpressing *PrDGAT1*, *PrPDAT1-2*, and *PrPDAT2*. Compared with the P19 control, the C18:3 composition of *PrDGAT1*- and *PrPDAT2*-expressing leaves increased by 5.3 and 10.6%, respectively, while the C16:0 SFA composition of *PrPDAT2*-expressing leaves decreased by 16.2% (Fig. 6E, Supplementary Data Table S2). In contrast, the level of C18:3 PUFA in *PrPDAT1-2*-expressing leaves decreased but that of C18:0 and C18:1 increased, compared with the P19 control (Fig. 6E). Despite the increased oil content of leaves expressing *PrDGAT2*, FA composition remained unaltered, with only a slight increase in the proportions of C18:2 and C18:3 (Fig. 6E). It is noteworthy that these analyses were generally consistent with the substrate specificities of PrDGATs and PrPDATs determined in yeast. PrDGAT1 increased the C18:3 composition to a relatively lower extent, accompanied by a slight increase in C18:1, probably due to its wide range of FA preferences. Although substrate preference assays showed that PrDGAT2 could transfer C18:2 and C18:3, its expression in tobacco leaves slightly increased the composition of C18:2 and C18:3, which indicates that it may have a weak role in ALA accumulation. In contrast, *PrPDAT2* expression in tobacco leaves increased the C18:3 composition to a greater extent, further confirming its unique preference for C18:3. Moreover, *PrPDAT1-2* transgenic tobacco leaves showed a significant increase in C18:1 composition, further confirming the ability of PrPDAT1-2 to transfer C18:1. Overall, *PrDGAT1*, *PrPDAT1-2*, and *PrPDAT2* expression in tobacco can alter FA composition; particularly, PrDGAT1 and PrPDAT2 can markedly increase the C18:3 proportion.

### Overexpressing *PrDGAT*s and *PrPDAT*s increases seed oil yield and alters the fatty acid profile in *A. thaliana* seeds

Given that *PrDGAT*s and *PrPDAT*s are predominantly expressed in seeds, we further investigated their role in stably transformed *Arabidopsis* seeds. 35S promoter-driven *PrDGAT* and *PrPDAT* expression constructs were introduced into Col-0 *Arabidopsis* and the three *T*_3_ homozygous transgenic lines with the highest gene transcript levels were selected for further analysis (Supplementary Data Fig. S4).

**Figure 7:**
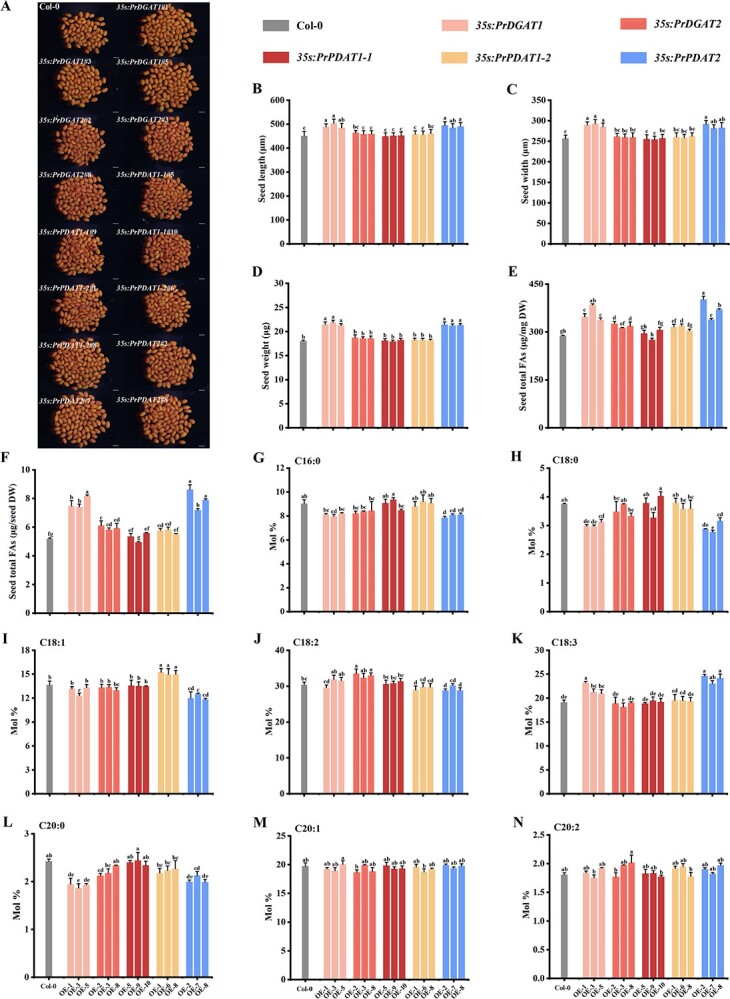
PrDGATs and PrPDATs elevate seed oil yield and alter the FA profile in transgenic *Arabidopsis* seeds. (A) Mature seed phenotypes of Col-0 and transgenic plants. Bars = 500 μm. (B and C) Seed size (B) and seed dry weight (C) of Col-0 and transgenic plants. (D and E) Total FA content (D) and total FA content (μg/dry seed) (E) in Col-0 and transgenic seeds. (F–M) Compositions of C16:0 (F), C18:0 (G), C18:1 (H), C18:2 (I), C18:3 (J), C20:0 (K), C20:1 (L), and C20:2 (M) in Col-0 and transgenic seeds. DW, dry weight. Values are mean ± standard deviation (*n* = 3). Different letters indicate significant difference at *P* < .05, as determined by Tukey’s post-test.

As shown in Fig. 7A, transgenic *Arabidopsis* seeds overexpressing *PrDGAT1* or *PrPDAT2* showed a marked increase in size (Fig. 7B and C) and dry weight (Fig. 7D) compared with wild-type (WT) seeds. However, these phenotypic changes were not observed in *PrDGAT2*, *PrPDAT1-1*, and *PrPDAT1-2* transgenic seeds. We further examined the seed oil content and found that *PrDGAT1*, *PrDGAT2*, *PrPDAT1-2*, and *PrPDAT2* transgenic *Arabidopsis* seeds showed increased oil levels compared with WT, whereas *PrPDAT1-1* transgenic *Arabidopsis* seeds did not, consistent with our results in tobacco (Fig. 7E). Specifically, *PrDGAT1* and *PrPDAT2* expression resulted in a more considerable increase in seed oil content, with their transgenic seeds having ~17.4–33.5 and ~17.5–37.4% higher total FA levels than WT, respectively (Fig. 7E, Supplementary Data Table S2). In addition, *PrDGAT2* and *PrPDAT1-2* expression caused a relatively modest increase in seed oil levels, with transgenic seeds having ~8.6–13.1 and ~5.4–10.8% higher total FA levels than WT, respectively (Fig. 7E). Consistently, the total FAs per seed of *PrDGAT1*, *PrDGAT2*, *PrPDAT1-2*, and *PrPDAT2* transgenic lines were significantly higher (Fig. 7F). These data suggest that PrDGAT1, PrDGAT2, PrPDAT1-2, and PrPDAT2 all play a part in seed oil synthesis. In addition, *PrDGAT1* and *PrPDAT2* expression in tobacco and *Arabidopsis* induced a relatively higher oil content (Fig. 6D and 7E), indicating that PrDGAT1 and PrPDAT2 may play a more important role in oil synthesis.

To investigate whether *PrDGAT1*, *PrDGAT2*, *PrPDAT1-2*, and *PrPDAT2* expression changed the FA composition while promoting oil synthesis, we further analyzed the FA profile of transgenic seeds. Compared with WT, *PrDGAT1*, *PrPDAT1-2*, and *PrPDAT2* transgenic seeds showed changes in FA composition, whereas *PrDGAT2* and *PrPDAT1-1* expression did not affect FA composition. *PrDGAT1* expression resulted in a markedly increased C18:3 composition, which was ~109–121% of that in WT, and significantly decreased composition of C18:0 and C20:0 (Fig. 7H, K, and L). In addition, PrDGAT1 also elevated the C18:2 composition in transgenic seeds, but to a relatively low extent (Fig. 7J), which may be due to its wide range of FA preferences. Consistent with the transfer preference for C18:1 of PrPDAT1-2 observed in H1246, *PrPDAT1-2* expression significantly increased the C18:1 proportion (Fig. 7I). *PrPDAT2* overexpression induced global FA composition changes in transgenic seeds, with a considerable increase in the C18:3 composition, which was ~120–128% of that in WT (Fig. 7K), and a decrease in the proportion of SFAs (C16:0, C18:0, and C20:0), C18:1, and C18:2. Clearly, these results confirm the unique transfer preference of PrPDAT2 for C18:3, which may determine its predominant role in ALA accumulation. Overall, our data suggest that PrDGAT1, PrPDAT1-2, and PrPDAT2 incorporate different FAs into seed oil and indicate a dominant role of PrPDAT2 in ALA accumulation.

### Lower *PrDGAT* and *PrPDAT* expression in *P. rockii* reduces oil content and affects fatty acid composition

**Figure 8:**
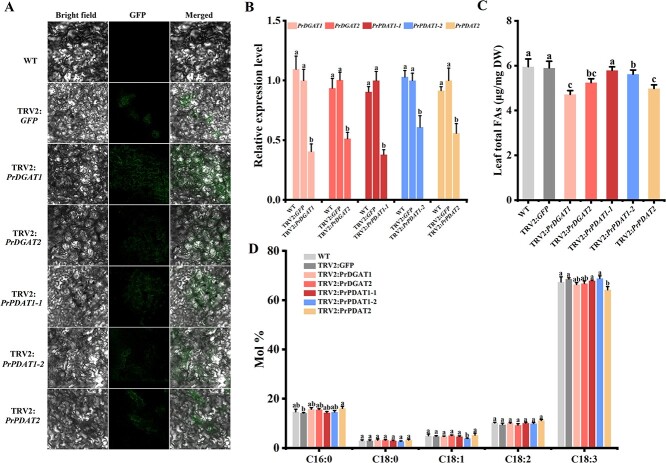
Reduced expression of *PrDGAT*s and *PrPDAT*s in *P. rockii* reduces oil content and affects FA composition. (A) Images of *P. rockii* leaves infiltrated with TRV2-*GFP* or TRV2- *PrDGAT*s/*PrPDAT*s under confocal microscopy at 6 dpi. Bars = 50 μm. (B) Silencing efficiency of *PrDGAT*s and *PrPDAT*s examined using RT–qPCR at 14 dpi. (C) Total FA contents in WT, TRV2:*GFP* control, and *PrDGAT*/*PrPDAT*-silenced leaves. (D) Fatty acid composition in WT, TRV2:*GFP* control, and *PrDGAT*/*PrPDAT*-silenced leaves. DW, dry weight. Values are mean ± standard deviation (*n* = 3). Different letters indicate significant difference at *P* < .05, as determined by Tukey’s post-test.

To further examine the role of *PrDGATs* and *PrPDATs* in tree peony, we reduced their endogenous transcription levels using virus-induced gene silencing (VIGS). As shown in Fig. 8A, GFP was detected on the leaves of gene silencing lines and TRV2:*GFP* control but not on WT. Moreover, *TRV1* and *TRV2* transcript accumulation was detected in inoculated leaves, but not in WT leaves (Supplementary Data Fig. S5). The quantification of endogenous expression of *PrDGAT*s or *PrPDAT*s showed that the transcriptional levels of the corresponding genes in the silenced lines were significantly lower than those in the WT and TRV2:*GFP* lines (Fig. 8B).

Given the reduced transcript levels of *PrDGAT*s or *PrPDAT*s in the silenced lines, we further determined the oil content in the leaves. Silencing of *PrDGAT*s or *PrPDAT*s, except for *PrPDAT1-1*, significantly reduced the oil content in the leaves. More specifically, *PrDGAT1*- and *PrPDAT2*-silenced plants showed the highest decrease in total FA content, which was ~80 and ~ 84% of the *TRV2:GFP* control, respectively (Fig. 8C, Supplementary Data Table S2), while *PrDGAT2*- and *PrPDAT1-2*-silenced plants showed a relatively modest decrease in total FA content (Fig. 8C). Thus, we can conclude that PrDGAT1, PrDGAT2, PrPDAT1-2, and PrPDAT2 promote oil synthesis. These data are consistent with the transgene assays in tobacco and *Arabidopsis*, further indicating that PrDGAT1 and PrPDAT2 have a stronger implication in oil synthesis.

As shown in Fig. 8D, the FA composition of *PrDGAT1*- and *PrDGAT2*-silenced lines did not change, although both showed a slight decrease in C18:3 composition compared with the TRV2:*GFP* control. However, *PrPDAT1-2* silencing resulted in a significantly lower C18:1 composition: ~82% of that in the TRV2:*GFP* control (Fig. 8D, Supplementary Data Table S2). Moreover, silencing *PrPDAT2* significantly decreased the C18:3 ratio to ~93% of that in the TRV2:*GFP* control but markedly increased C16:0 composition (Fig. 8D). We observed that the lower *PrDGAT1* expression did not significantly decrease C18:3 composition but slightly decreased C18:1, which may be due to its wide range of FA transfer preferences. In addition, silencing *PrPDAT2* significantly reduced the C18:3 composition, which could be attributed to its unique transfer preference for C18:3. In conclusion, this assay helps further demonstrate that PrPDAT2 plays a predominant role in ALA accumulation.

### Conclusions

In the present study, a total of 760 lipid molecules were identified in *P. rockii* seeds and categorized into six classes, including glycerolipids, glycerophospholipids, saccharolipids, sphingolipids, prenol lipids, and fatty acyls. These lipid compounds were further categorized into 27 subclasses, with TAGs being the most abundant and having the most diverse variety among them. Both TAG species and abundance increased during seed development, peaking at seed maturity. Furthermore, FA localization analysis of TAG showed that C18:3 was the predominant FA at the TAG *sn*-3 position throughout seed development, with >80% PUFA at the TAG *sn*-3 position at seed maturity. A total of five acyltransferases genes involved in FA transfer to the TAG *sn*-3 position were isolated from *P. rockii* seeds, including *PrDGAT1*, *PrDGAT2*, *PrPDAT1-1*, *PrPDAT1-2*, and *PrPDAT2*. Functional complementation analysis of these genes indicated their roles in TAG synthesis, except for PrPDAT1-1, and their different FA transfer preferences. Biological experiments further revealed that PrDGAT1, PrDGAT2, PrPDAT1-2, and PrPDAT2 promote oil accumulation in both transgenic *N. benthamiana* leaves and stable transgenic *A. thaliana* seeds, with PrPDAT2 preferentially accumulating ALA into oil, as confirmed by gene silencing in *P. rockii* leaves. Overall, PrDGAT1 and PrPDAT2 could promote ALA accumulation in seed oil, with PrPDAT2 playing a predominant role. Our findings provide novel functional evidence of the involvement of *PrDGAT1* and *PrPDAT2* in enhancing ALA yield in *P. rockii* and other oilseed crops.

## Materials and methods

### Plant materials and growth conditions

*Paeonia rockii* seeds were collected from the Northwest A&F University at 20, 40, 60, 80, and 100-DAP, denoted as S1, S2, S3, S4, and S5 stages, respectively. The collected seeds and other organs were stored at −80°C for further assays. For the VIGS test, 2-year-old *P. rockii* plants were employed. The plants used in this study were cultivated in a climate room at 22°C.

### Chemicals

Methanol (MeOH), chloroform, and formic acid were LC–MS grade. Acetonitrile (ACN), ammonium formate (AF), methyl *tert*-butyl ether (MTBE), and isopropanol (ISO) were acquired from Merck. Standard mixtures for lipid determination contained LPC 18:1 (d7), PC 15:0–18:1 (d7), PG 15:0–18:1 (d7), PA 15:0–18:1 (d7), PS 15:0–18:1 (d7), PE 15:0–18:1 (d7) PI 15:0–18:1 (d7), SM d18:1–18:1 (d9), CE 16:0 (d7), MG 18:1 (d7), DAG 15:0–18:1 (d7), and TAG 16:0–18:0–16:0 (d5); all were compliant with the SPLASH^®^ LIPIDOMIX® Mass Spec Standard.

### Total lipid extraction

Twenty milligrams of freeze-dried sample powder was collected in a 2-ml tube along with a steel ball. Subsequently, we added 1 ml of lipid extract (MTBE:MeOH = 3:1, v/v) and vibrated for 30 minutes. Then, 300 μl of ultrapure water was added, and the sample was vibrated for 1 minute and incubated at 4°C for 10 minutes. Next, the sample was centrifuged for 4 minutes to retrieve the supernatant, from which 300 μl was added to a tube and evaporated completely at 20°C. Subsequently, 200 μl of the solution (ACN:ISO = 1:1, v/v) was added to re-solubilize samples and the sample was centrifuged for 10 minutes. One hundred and twenty microliters of supernatant was added in a phial for further LC–MS analysis. To examine the consistency and stability of the analysis, we added 20 μl of each sample into the QC sample.

### UPLC–MS analysis

The data collection instruments were mainly the UPLC system (ExionLC™ AD, Sciex) and MS/MS system (QTRAP^®^ 6500+, Sciex). UPLC system conditions included a C30 column. In addition, the mobile phase in positive ion mode consisted of solvent A (ACN:water = 60:40, v/v, with 0.1% formic acid and 10 mM AF) and solvent B (ACN:ISO = 10:90, v/v, with 0.1% formic acid and 10 mM AF). The gradient elution program was set as described previously [33]. The column temperature was held at 45°C, the flow rate was 0.35 ml/minute, and the sample volume was 2 μl. The MS parameters were as follows: electrospray ionization temperature 500°C, 5500 V in positive ionization mode, and −4500 V in negative ionization mode. For the ion source, gas 1 was 45 psi, gas 2 was 55 psi, and curtain gas was 35 psi.

### Data processing

The MS-DIAL software was used to compare the MS/MS spectrum with the reference lipid compounds in the LipidBlast database to identify the lipid species based on the following criteria: mass accuracy (precursor ion, <0.01 Da; fragment ion, <0.05 Da), isotopic pattern (variance, <10%), retention time tolerance (5 min), MS/MS accuracy mass tolerance (0.05 Da), and identification cutoff value (80%). A lipid quantification method using MasterView software and further applied to MultiQuant software was established for in-depth quantitative analysis. Lipid content was determined with respect to an internal standard using the analyte relative peak area.

### Bioinformatics analysis

NCBI CD-Search was used to analyze the conserved domains of *PrDGAT*s and *PrPDAT*s. Amino acid sequence alignment was conducted with ClustalX software. The neighbor-joining method was used to generate phylogenetic trees in MEGA 7.0.

### Expression analysis

RNA extraction and reverse transcription were performed as previously described [34]. RT–PCR was used to examine *PrDGAT* and *PrPDAT* transcripts. RT–qPCR was used to analyze relative gene expression. The *18S* gene, *NbL23*, and *Atactin7* were used as internal controls to normalize gene expression using the 2^-ΔΔCT^ method. The primers are listed in Supplementary Data Table S3. Three biological replicates were performed for each assay.

### Subcellular localization analysis

The coding sequences of *PrDGAT*s and *PrPDAT*s without stop codons were cloned into pCAMBIA2300-GFP to obtain the constructs. The constructs and *NtERMP1*-*RFP*, an ER marker, were transformed into GV3101 and injected into tobacco leaves [35]. After 48 hours post-infiltration, fluorescence was examined using a confocal laser scanning microscope.

### Heterologous expression in *S. cerevisiae* mutants

The coding sequences of *PrDGAT*s and *PrPDAT*s were cloned into pESC-URA to obtain the constructs. TAG-deficient H1246 competent cells were prepared and transformed with the said constructs using S.c. EasyComp™ kits (Invitrogen). VfDGAT1, isolated from *Vernicia fordii*, was previously shown to complement H1246 to restore TAG synthesis. H1246 carrying *VfDGA1* and the empty pESC-URA vector were used as positive and negative controls, respectively. Transformants were screened on medium deficient in uracil (SD-URA). Transformants were cultured overnight in liquid SD-URA with 2% raffinose and inoculated with liquid SDGG-URA medium containing 2% galactose and 1% raffinose at a starting OD_600_ of 0.1 to induce gene expression. Cultures with a starting OD_600_ of 0.1 were then transferred to an SDGG-URA medium containing 0.2% Tergitol and 1.0 mM free FAs. Cultures were shaken at 28°C and cell density was examined at different time points.

### *Agrobacterium tumefaciens-*mediated overexpression assay in *N. benthamiana* and *A. thaliana*

For the transient overexpression assay in *N. benthamiana* leaves, the coding sequences of *PrDGAT*s and *PrPDAT*s were cloned into pB110 to obtain the constructs. To promote the expression of *PrDGAT*s and *PrPDAT*s, GV3101 suspensions containing the constructs and P19 silencing inhibitors were mixed in equal volumes and injected into leaves as described previously [34]. The LDs were stained and confocal images were acquired as described previously [34].

For stable overexpression in *A. thaliana* seeds, pCAMBIA1300 constructs containing the coding sequences of *PrDGAT*s and *PrPDAT*s were transformed into *Arabidopsis* inflorescences. Transgenic lines were obtained and identified as described previously [34]. Then, transgenic plants were grown to T3. Seed phenotypes were collected under a microscope.

### Virus-induced gene silencing assay

To obtain TRV2:*PrDGAT* and TRV2:*PrPDAT* constructs, non-conserved fragments of *PrDGAT*s and *PrPDAT*s of ~300 bp length were cloned into TRV2:*GFP*. *Paeonia rockii* leaves were injected with a 1:1 GV3101 suspension including TRV1 and TRV2. Untreated leaves and TRV2:*GFP* inoculation leaves served as controls. GFP was examined at 6 dpi using a confocal laser scanning microscope (Leica; SP8). At 14 dpi, 10 individual seedling samples were collected from each group to examine the transcript levels of the target genes and freeze-dried for oil analysis.

### Fatty acid quantification

A total of 50 mg of *N. benthamiana* and *P. rockii* leaves or 10 mg of naturally dried *A. thaliana* seeds were used for FA extraction as described previously [34]. FAs were analyzed following extraction using a gas chromatography system (Agilent; 8890). Three biological replicates were performed for each assay. To normalize the FA concentrations of *A. thaliana*, *N. benthamiana*, and *P. rockii*, C13:0 and C17:0 were used as internal controls, respectively.

## Acknowledgements

We thank Man Zhang (Beijing Forestry University) for her guidance in writing, reviewing, and editing. This work was supported by the National Natural Science Foundation of China (31971690, 31901357, and 31972453).

## Author contributions

W.Y., investigation, writing, data curation. Z.X., investigation, data curation. L.X. and Y.Z., investigation. YL.Z. and L.N., writing, reviewing, and editing. Q.Z., supervision.

## Data availability

All data supporting the conclusions of this work are present in the paper or its Supplementary material files. Sequence data from this article can be obtained in the TAIR/GenBank data libraries under accession numbers AtDGAT1 (AT2G19450), AtDGAT2 (AT3G51520), AtPDAT (AT5G13640), PrDGAT1 (OQ222883), PrDGAT2 (OQ784271), PrPDAT1-1 (OQ784272), PrPDAT1-2 (OQ784273), and PrPDAT2 (OQ222884).

## Conflict of interest statement

None declared.

## Supplementary Data

Supplementary data are available at *Horticulture Research* online.

## Supplementary Material

Web_Material_uhad106
