# Supplementary material for: Integrative lipidomics profile uncovers the mechanisms underlying high-level α-linolenic acid accumulation in Paeonia rockii seeds
Source: Hortic Res. 2023 May 15;10(7):uhad106. doi: 10.1093/hr/uhad106 (PMC10419846; doi:10.1093/hr/uhad106)
Supplement: Web_Material_uhad106 [file web_material_uhad106.zip › Supplementary Figures.docx]

**Supplementary Figures**

**
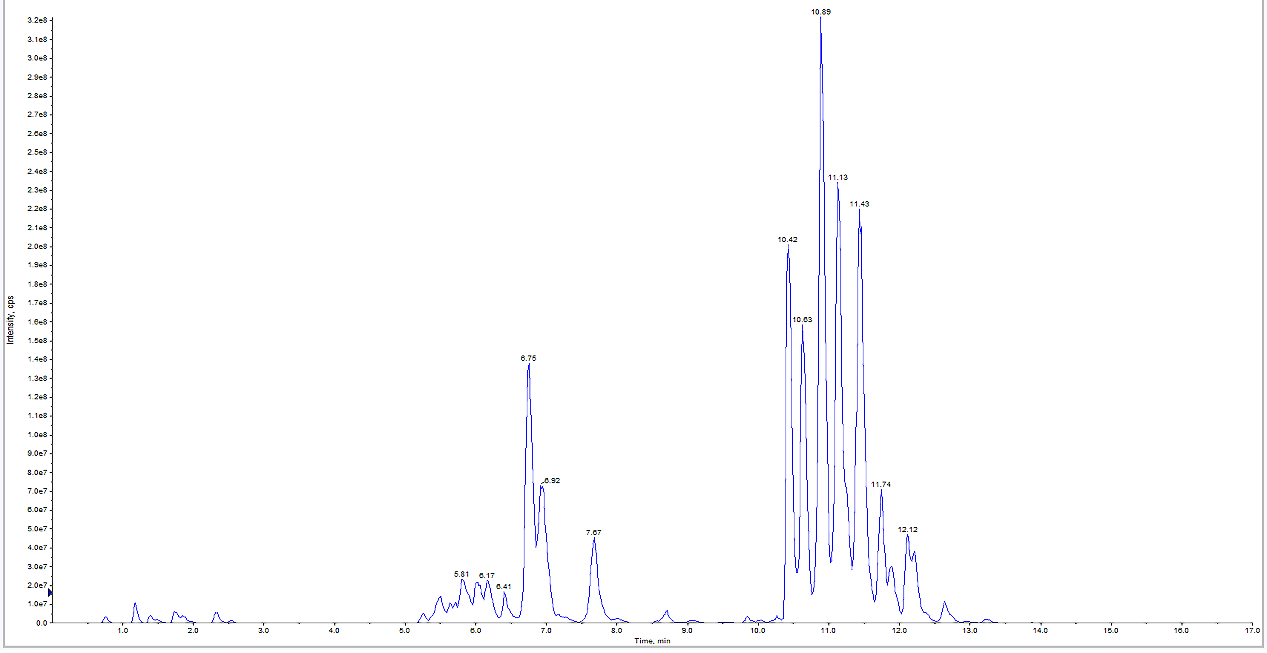
**

**Figure S1.** The spectrum of QC samples.

**
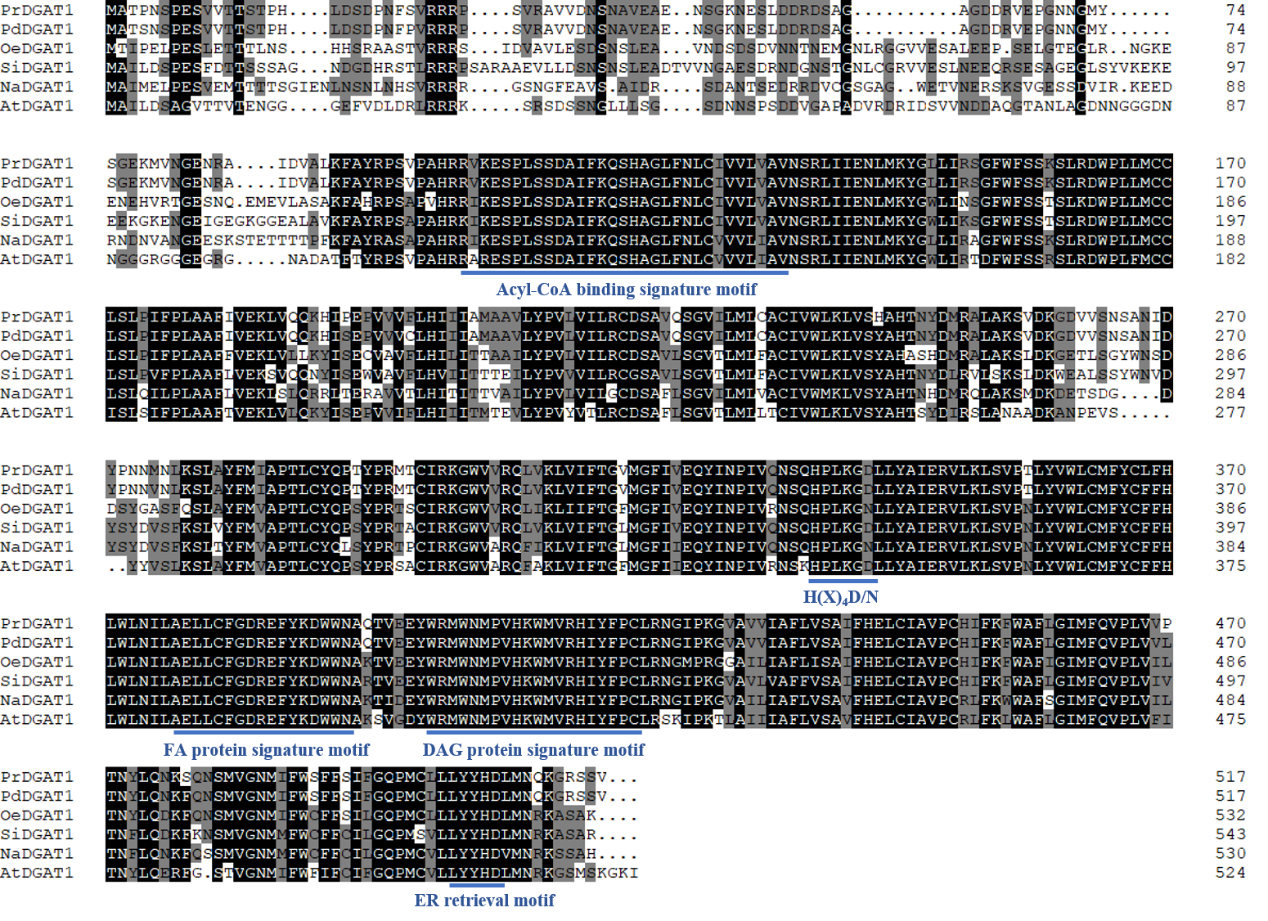
(A)**

**(B)**


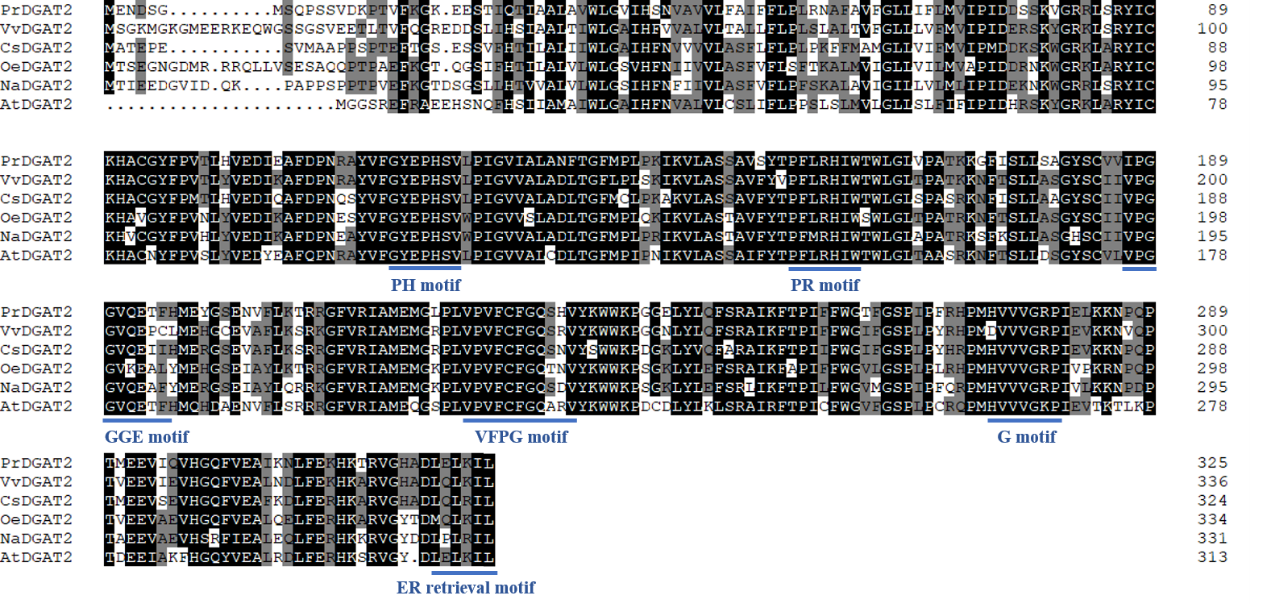


**(C)**

**
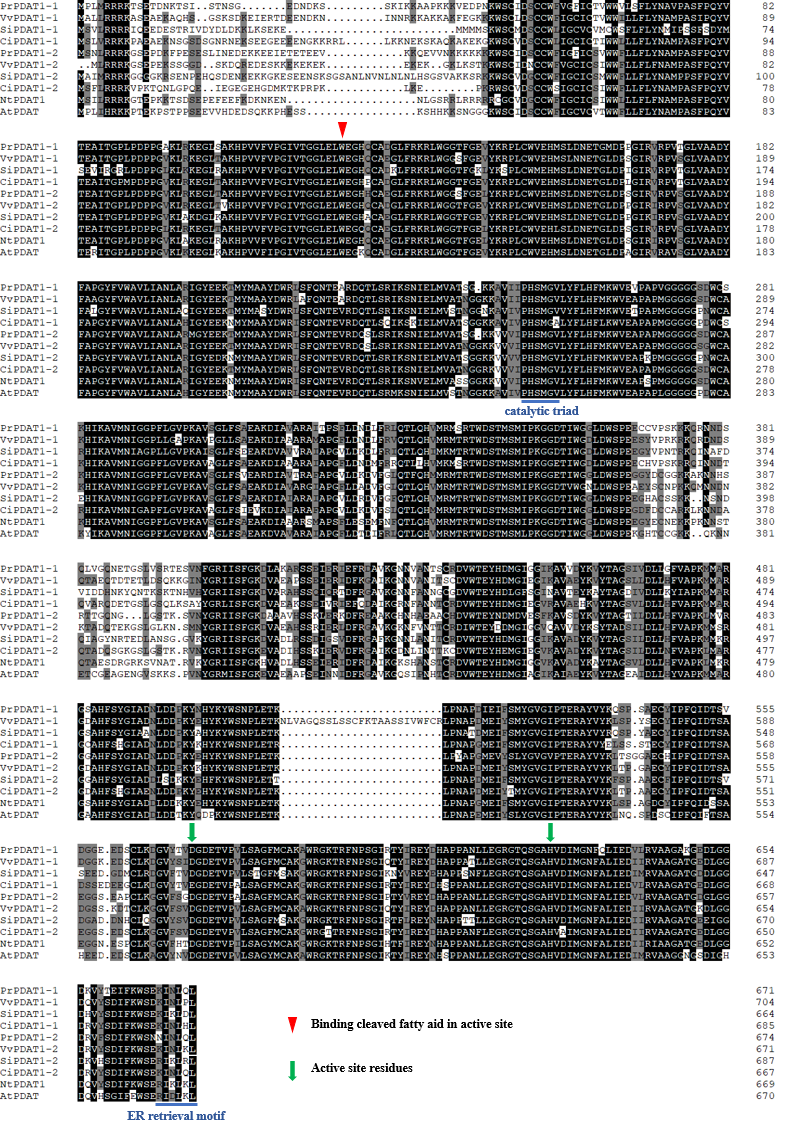
**

**(D)**

**
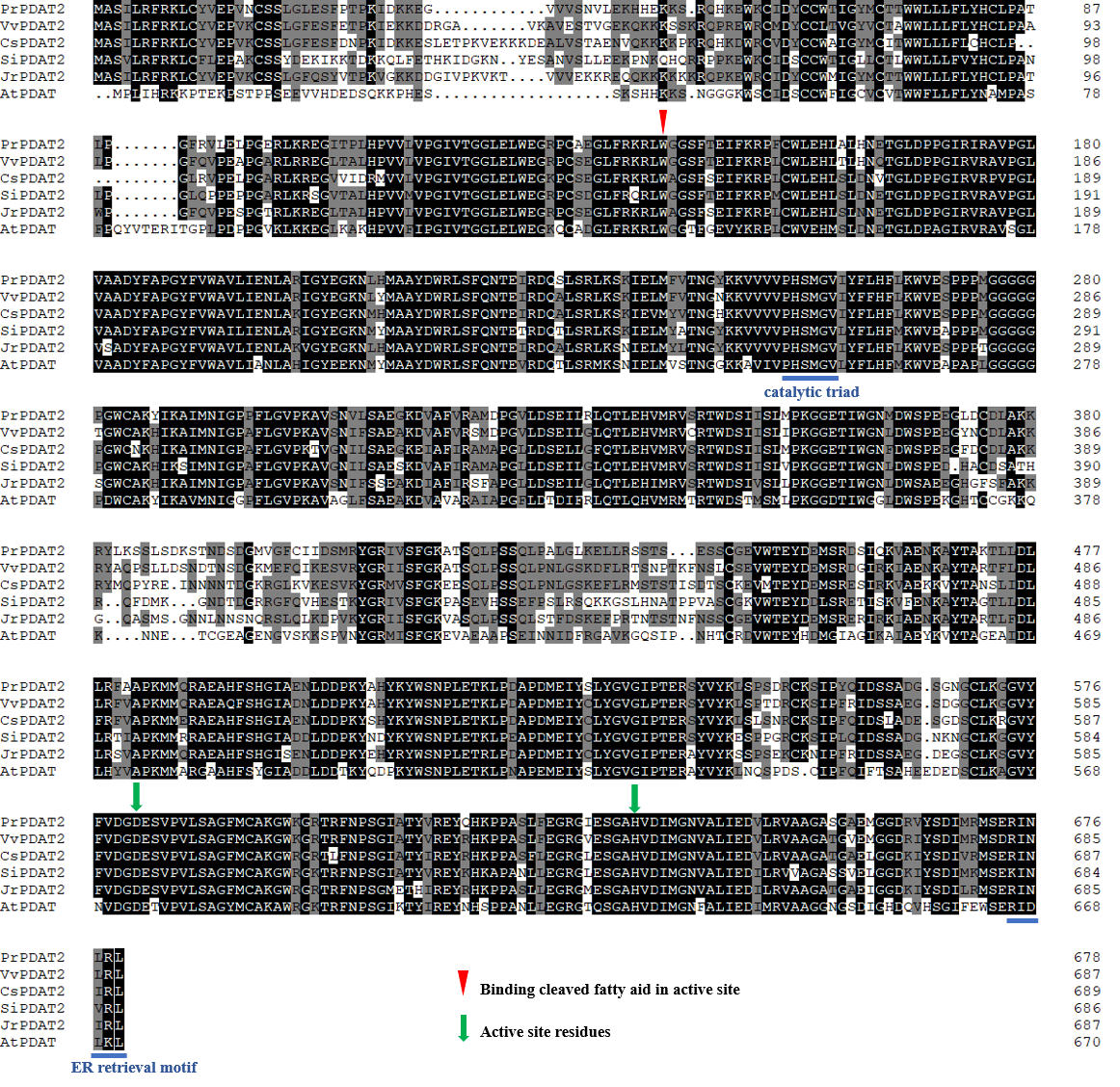
**

**Figure S2.** Sequence alignment of PrDGAT1 **(A)**, PrDGAT2 **(B)**, PrPDAT1-1, PrPDAT1-2 **(C)**, and PrPDAT2 **(D)** proteins. Pr: Paeonia rockii; Pd: Paeonia delavayi; Oe: Olea europaea; Si: Sesamum indicum; Na: Nicotiana attenuata; Vv: *Vitis vinifera*; Cs: *Camellia sinensis*; Na: *Nicotiana attenuata*; Ci: *Carya illinoinensis*; Nt: *Nicotiana tabacum*; At: *Arabidopsis thaliana*. The conserved domains are marked underlined.

**
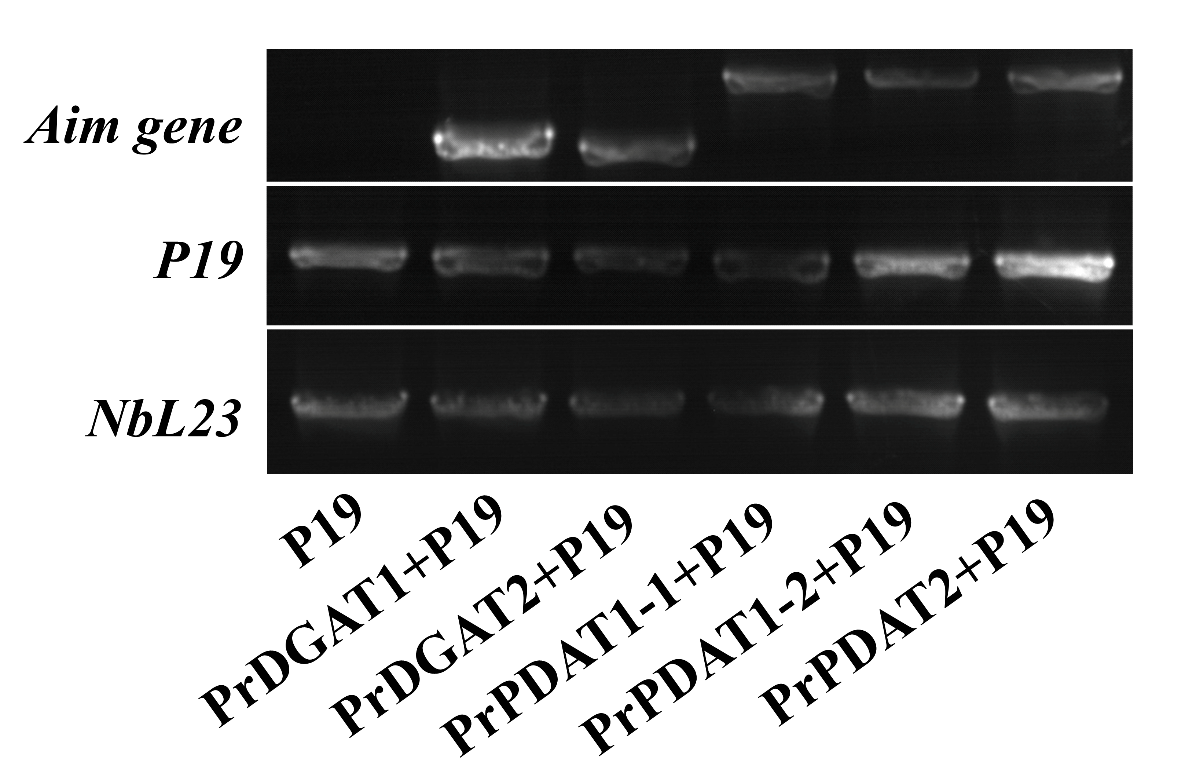
**

**Figure S3.** RT-PCR analysis of *PrDGATs* and *PrPDATs* expressed in tobacco leaves.

**
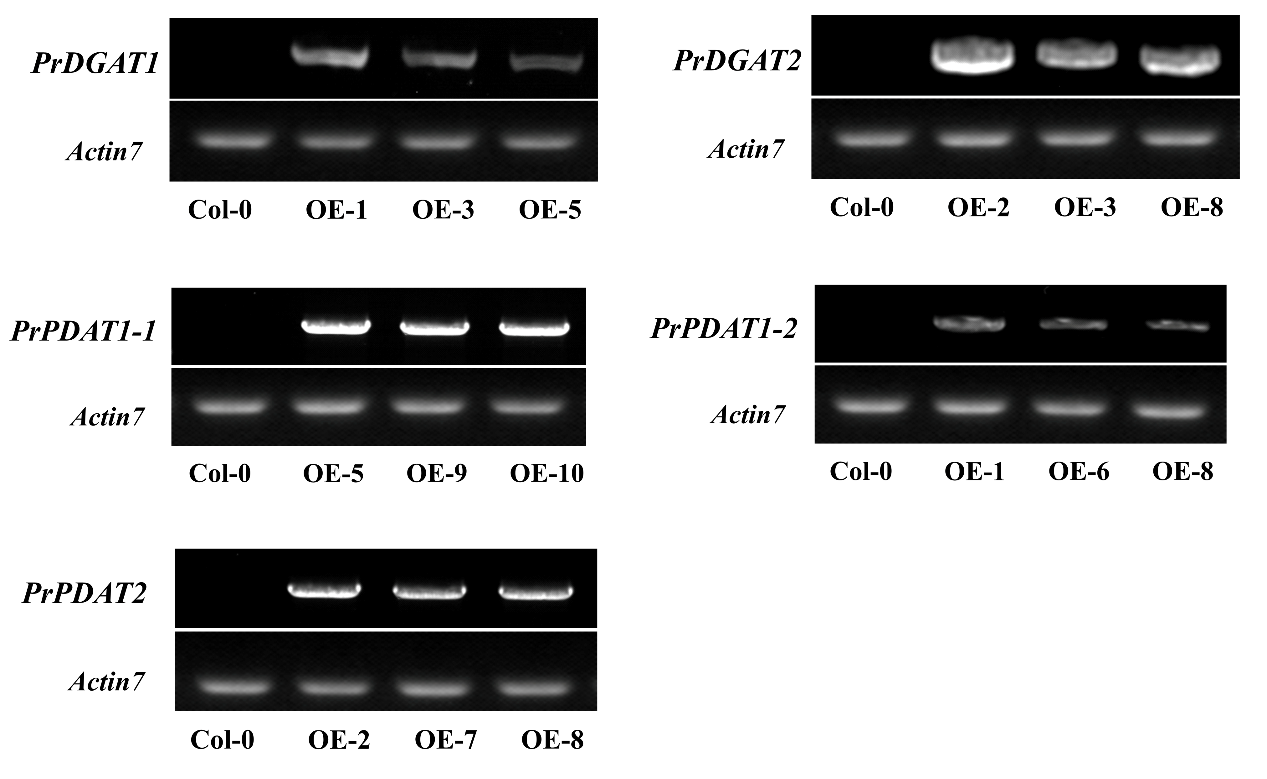
**

**Figure S4.** RT-PCR analysis of *PrDGATs* and *PrPDATs* expressed in transgenic Arabidopsis seeds.

**
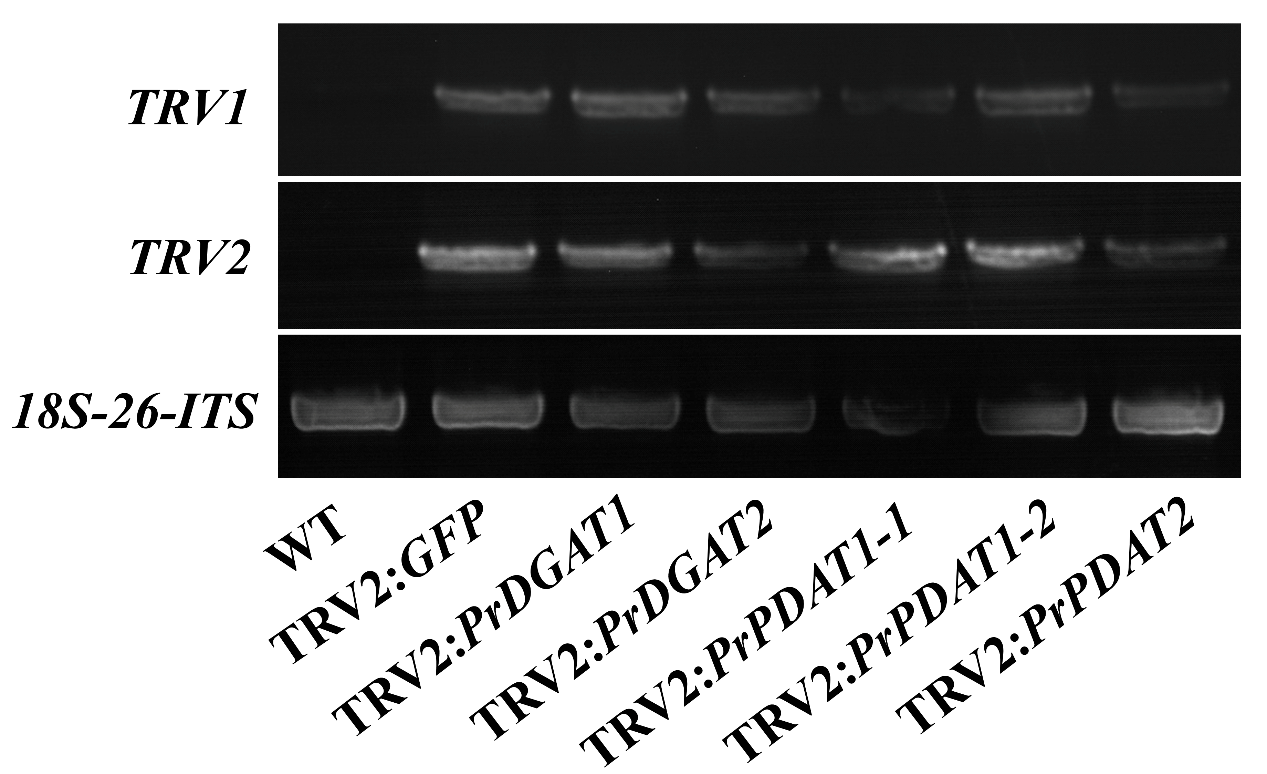
**

**Figure S5.** RT-PCR of *TRV1* and *TRV2* levels in *P. rockii* leaves.

**Appendix S1.** Nucleotide and amino acid sequences of PrDGAT1 **(A)**, PrDGAT2 **(B)**, PrPDAT1-1 **(C)**, PrPDAT1-2 **(D)**, and PrPDAT2 **(E)**. The conserved domains are marked underlined.

**(A)**

**>*PrDGAT1* (1554bp)**

ATGGCGACCCCGAATTCGCCGGAAAGTGTAGTCACTACATCGACCCCTCACCTCGACTCAGATCCCAACTTCTCTGTTCGGAGGAGACCGAGTGTCAGGGCGGTTGTTGATAACTCGAACGCGGTTGAAGCTGAGAATTCGGGTAAAAATGAATCGCTGGACGATAGGGACTCAGCCGGGGCTGGCGATGATAGGGTGGAACCGGGGAATAATGGAATGTACAGTGGTGAGAAGATGGTAAATGGTGAGAATCGAGCGATCGATGTCGCCTTGAAATTCGCTTATCGGCCTTCTGTTCCAGCTCATCGGAGAGTGAAAGAGAGTCCTCTCAGTTCTGACGCCATTTTCAAACAGAGCCATGCGGGTCTCTTCAACCTTTGTATAGTTGTGCTTGTTGCAGTAAACAGCCGTCTTATTATTGAGAATCTCATGAAGTATGGTTTGTTAATCAGGTCCGGTTTTTGGTTTAGTTCAAAATCATTGAGAGATTGGCCCCTTCTCATGTGCTGTCTATCTCTCCCGATTTTTCCTCTTGCCGCATTTATAGTTGAAAAGTTGGTGCAACAAAAGCATATACCTGAACCGGTTGTTGTCTTCCTCCATATAATAATTGCCATGGCTGCAGTTTTGTATCCAGTTTTAGTGATTCTCAGGTGTGAT0.TCTGCTGTCCAATCTGGTGTCATACTAATGCTCTGTGCTTGCATTGTGTGGTTAAAATTGGTATCGCATGCGCACACAAACTATGATATGAGAGCACTTGCTAAGTCAGTTGATAAGGGGGATGTCGTGTCCAATTCTGCGAATATTGATTACCCAAACAACATGAACTTGAAGAGTTTGGCATACTTCATGATTGCTCCCACGTTATGTTACCAGCCAACCTATCCTCGTATGACATGCATCCGAAAGGGTTGGGTAGTTCGTCAACTTGTCAAGTTGGTAATATTTACCGGAGTTATGGGATTTATTGTAGAACAATATATTAATCCTATTGTCCAGAACTCTCAGCATCCTTTGAAAGGGGATCTTTTATATGCTATAGAGAGGGTTTTGAAGCTTTCGGTACCAACATTATATGTGTGGCTTTGCATGTTCTACTGCCTTTTTCACCTCTGGTTAAATATACTAGCTGAACTTCTTTGTTTTGGGGATCGTGAGTTTTACAAAGATTGGTGGAATGCGCAAACAGTTGAGGAGTATTGGAGAATGTGGAACATGCCTGTTCATAAATGGATGGTTCGCCATATATATTTTCCATGCTTACGGAATGGGATACCCAAGGGAGTTGCTGTTGTGATTGCCTTTTTAGTATCTGCCATATTTCACGAGTTATGCATTGCCGTCCCTTGCCACATATTCAAGTTTTGGGCGTTCTTAGGAATTATGTTTCAGGTTCCCTTGGTTGTGCCCACAAATTACCTGCAAAATAAGTCTCAAAATTCTATGGTGGGGAATATGATATTCTGGTCATTTTTTAGCATATTTGGTCAACCCATGTGTCTGCTGCTTTATTATCATGACTTGATGAACCAAAAAGGAAGAAGTAGTGTATAA

**>PrDGAT1 (517aa)**

MATPNSPESVVTTSTPHLDSDPNFSVRRRPSVRAVVDNSNAVEAENSGKNESLDDRDSAGAGDDRVEPGNNGMYSGEKMVNGENRAIDVALKFAYRPSVPAHRRVKESPLSSDAIFKQSHAGLFNLCIVVLVAVNSRLIIENLMKYGLLIRSGFWFSSKSLRDWPLLMCCLSLPIFPLAAFIVEKLVQQKHIPEPVVVFLHIIIAMAAVLYPVLVILRCDSAVQSGVILMLCACIVWLKLVSHAHTNYDMRALAKSVDKGDVVSNSANIDYPNNMNLKSLAYFMIAPTLCYQPTYPRMTCIRKGWVVRQLVKLVIFTGVMGFIVEQYINPIVQNSQHPLKGDLLYAIERVLKLSVPTLYVWLCMFYCLFHLWLNILAELLCFGDREFYKDWWNAQTVEEYWRMWNMPVHKWMVRHIYFPCLRNGIPKGVAVVIAFLVSAIFHELCIAVPCHIFKFWAFLGIMFQVPLVVPTNYLQNKSQNSMVGNMIFWSFFSIFGQPMCLLLYYHDLMNQKGRSSV.

**(B)**

**>*PrDGAT2* (981bp)**

ATGGAGAACGATTCGGGAATGTCTCAGCCGTCGTCCGTGGATAAACCGACGGTATTCAAGGGAAAAGAAGAGTCCACAATACAGACAATTGCAGCTTTGGCAGTATGGCTAGGCGTCATCCATTCCAATGTCGCTGTAGTCCTCTTCGCGATATTCTTCCTTCCTCTTCGCAACGCCTTCGCGGTGTTTGGATTGCTTATATTTTTAATGGTTATACCAATCGACGATAGCAGCAAAGTAGGTCGAAGGTTATCCAGGTATATATGTAAGCATGCCTGTGGTTATTTTCCGGTGACTCTACATGTGGAGGATATAGAGGCCTTTGATCCAAATCGTGCATATGTCTTTGGTTATGAGCCCCATTCAGTTTTGCCAATTGGTGTCATTGCACTTGCCAACTTTACGGGTTTCATGCCTCTCCCAAAAATAAAGGTCCTCGCAAGTAGTGCTGTGTCCTACACACCATTCTTGAGGCATATATGGACATGGTTGGGTCTTGTACCTGCAACAAAGAAAGGTTTTATTTCCCTTTTGTCAGCTGGTTATAGTTGCGTCGTAATACCTGGTGGAGTGCAAGAAACATTTCATATGGAGTATGGTTCTGAGAATGTTTTCCTGAAGACGAGAAGAGGATTTGTTCGTATTGCCATGGAGATGGGCCTACCCCTAGTTCCAGTTTTCTGTTTTGGCCAGTCACATGTCTATAAGTGGTGGAAGCCTGGCGGGGAACTGTATTTGCAATTTTCTAGAGCCATCAAATTCACTCCAATTTTCTTTTGGGGAACATTTGGATCTCCAATCCCCTTTCGGCATCCAATGCACGTGGTTGTAGGAAGACCCATTGAGCTTAAGAAAAATCCACAACCCACTATGGAAGAGGTGATTCAAGTACACGGTCAGTTCGTTGAAGCAATAAAGAATCTGTTTGAAAAGCACAAAACGCGGGTTGGCCATGCCGATCTTGAATTGAAAATATTGTGA

**>PrDGAT2 (326aa)**

MENDSGMSQPSSVDKPTVFKGKEESTIQTIAALAVWLGVIHSNVAVVLFAIFFLPLRNAFAVFGLLIFLMVIPIDDSSKVGRRLSRYICKHACGYFPVTLHVEDIEAFDPNRAYVFGYEPHSVLPIGVIALANFTGFMPLPKIKVLASSAVSYTPFLRHIWTWLGLVPATKKGFISLLSAGYSCVVIPGGVQETFHMEYGSENVFLKTRRGFVRIAMEMGLPLVPVFCFGQSHVYKWWKPGGELYLQFSRAIKFTPIFFWGTFGSPIPFRHPMHVVVGRPIELKKNPQPTMEEVIQVHGQFVEAIKNLFEKHKTRVGHADLELKIL.

**(C)**

**>*PrPDAT1-1* (2019bp)**

ATGCCATTAATGCGACGGAGAAAAACATCTGAAACTGATAACAAAACTTCGATTTCAACAAATAGTGGAGAAGACAACGACAAGAGCAGCAAAATCAAGAAGGCTGCTCCGAAGAAGAAAGTTGAAGACCCAAATAAGTGGTCATGTATCGATAGCTGCTGTTGGTTCGTAGGGTTTATATGTACGGTGTGGTGGGTACTCTCGTTTCTATACAATGCGGTGCCGGCTTCGTTCCCTCAGTACGTCACGGAGGCCATCACAGGGCCGTTGCCAGACCCTCCAGGTGCGAAATTGCGTAAAGAAGGGTTGTCGGCAAAGCATCCGGTGGTGTTCGTGCCGGGTATTGTGACGGGTGGGCTTGAATTATGGGAAGGGCATCAGTGTGCGGATGGGTTGTTTCGGAAGCGGCTTTGGGGGGGCACATTTGGAGAAGTTTATAAAAGACCTTTATGCTGGGTAGAGCATATGTCACTGGACAATGAGACTGGAATGGATCCTCCTGGTATAAGGGTCAGGCCTGTCACTGGACTTGTAGCAGCAGATTACTTCGCTCCGGGCTATTTTGTATGGGCAGTTCTTATTGCCAATTTAGCTCGCATTGGGTATGAGGAAAAAACCATGTATATGGCTGCCTATGATTGGAGACTCTCATTTCAGAACACTGAGGCGCGGGATCAAACACTAAGTAGAATAAAAAGTAATATCGAACTGATGGTTGCTACAAGTGGAAAAAAAGCAGTTATCATTCCACATTCCATGGGCGTTTTGTACTTTTTGCATTTTATGAAGTGGGTTGAGGTTCCGGCTCCAGTGGGTGGTGGGGGTGGATCAGATTGGTGTTCTAAGCATATAAAGGCAGTGATGAACATTGGTGGACCATTTTTAGGTGTTCCAAAGGCCGTTTCTGGGCTTTTCTCTGCTGAAGCCAAAGATATAGCAGTTGCCAGAGCAATTACACCAAGCTTTTTGGATAATGATTTATTTCGGCTACAAACATTGCAACATGTGATGAGAATGAGCCGCACATGGGATTCAACCATGTCCATGATACCAAAAGGTGGCGATACTATTTGGGGTGGTCTTGACTGGTCACCTGAAGAATGTTGTGTTCCTAGCAAGAAAAAGCAAAGAAACAATGATAGTCAGCTTGTAGGGCAAAATGAGACTGGAAGTCTTGTTTCTCGAACAGAAAGTGTTAATTTTGGGAGGATTATTTCATTTGGGAAAGATCTAGCCAAGGCACGGTCATCTGAGATTGAGAGGATTGAATTTAGGGATGCTGTTAAGGGTAACAATGTTGCAAACACAAGCTGTCGTGATGTGTGGACGGAGTACCATGACATGGGAATTGGAGGCATTAAAGCTGTTGTCGACTACAAAGTTTACACTGCTGGGTCAATTGTGGATCTGCTCGGTTTTGTTGCTCCCAAAATGATGGCACGCGGCAGTGCTCATTTTTCGTATGGAATTGCTGACAACTTGGATGATCCAAAATACAACCACTATAAATATTGGTCAAATCCTTTGGAAACAAAATTACCAAATGCTCCTGACATTGAAATATTCTCGATGTATGGAGTTGGTATCCCAACTGAAAGAGCGTATGTTTACAAGCAATCTCCTTCTGCTGAATGCTACATTCCATTTCAGATCGATACATCAGTTGATGGTGGAGAGGAAGACAGCTGCCTAAAAGATGGAGTCTACACAGTCGACGGGGACGAGACAGTGCCTGTTTTAAGTGCAGGCTTCATGTGTGCAAAAGCTTGGCGTGGAAAAACCAGATTCAATCCTTCTGGGATTCGTACTTACATTCGGGAGTACGATCACGCTCCCCCAGCCAATCTTTTAGAAGGTCGGGGCACACAGAGTGGTGCACATGTGGATATAATGGGGAATTTTCAATTGATTGAGGATGTTTTAAGGGTAGCAGCTGGGGCTAAAGGTGAAGACTTGGGAGGTGATAAAGTGTACACTGAGATATTCAAGTGGTCGGAGAAGATCAATTTACAACTATAA

**>PrPDAT1-1 (672aa)**

MPLMRRRKTSETDNKTSISTNSGEDNDKSSKIKKAAPKKKVEDPNKWSCIDSCCWFVGFICTVWWVLSFLYNAVPASFPQYVTEAITGPLPDPPGAKLRKEGLSAKHPVVFVPGIVTGGLELWEGHQCADGLFRKRLWGGTFGEVYKRPLCWVEHMSLDNETGMDPPGIRVRPVTGLVAADYFAPGYFVWAVLIANLARIGYEEKTMYMAAYDWRLSFQNTEARDQTLSRIKSNIELMVATSGKKAVIIPHSMGVLYFLHFMKWVEVPAPVGGGGGSDWCSKHIKAVMNIGGPFLGVPKAVSGLFSAEAKDIAVARAITPSFLDNDLFRLQTLQHVMRMSRTWDSTMSMIPKGGDTIWGGLDWSPEECCVPSKKKQRNNDSQLVGQNETGSLVSRTESVNFGRIISFGKDLAKARSSEIERIEFRDAVKGNNVANTSCRDVWTEYHDMGIGGIKAVVDYKVYTAGSIVDLLGFVAPKMMARGSAHFSYGIADNLDDPKYNHYKYWSNPLETKLPNAPDIEIFSMYGVGIPTERAYVYKQSPSAECYIPFQIDTSVDGGEEDSCLKDGVYTVDGDETVPVLSAGFMCAKAWRGKTRFNPSGIRTYIREYDHAPPANLLEGRGTQSGAHVDIMGNFQLIEDVLRVAAGAKGEDLGGDKVYTEIFKWSEKINLQL.

**(D)**

**>*PrPDAT1-2* (2028bp)**

ATGTCCAACTTGAGACGCAGAAAGGGCTCTGAACCCGATAAATTTCCCGAGTCAGAATCTCTGATTAATGAAGATGAGAAAAAAGAGGAGATAGAGACAGAGACGGAGGAGGTGAAGAAACAAGAGGTGGTGAATAAAAAGAAGAAGAAAAAGAAGTGGTCTTGTATTGATAGCTGTTGTTGGTTTATTGGCTTTATTTGTTCGGTTTGGTGGTTCTTGTTGTTTCTTTACAATGCTATGCCGGCATCATTCCCTCAGTACGTCACGGAAGCCATTACAGGACCTTTGCCAGACCCGCCCGGCGTCAAATTGAGGAAAGAAGGGTTAACGGCCAAGCACCCAGTGGTTTTCGTACCTGGGATTGTTACAGGTGGACTTGAGTTGTGGGAAGGGCACCATTGTGCTGAGGGATTGTTCAGAAAGAGGCTTTGGGGTGGTTCATTTGGAGAACTTTACAAAAGACCCTTATGTTGGGTTGAGCACATGTCGTTGGATAATGAAACTGGATTGGATCCCTCTGGTATAAGGGTTAGGCCTGTATCTGGACTTGTTGCTGCCGATTACTTTGCACCAGGTTATTTTGTATGGGCAGTTTTAATTGCTAATTTGGCTCGCATGGGGTATGAGGAGAAGACCATGTATATGGCCGCATATGATTGGAGACTTTCATTTCAGAACACTGAGGTCAGGGACCAAAGTCTAAGTAGAATAAAGAGTAATATAGAGCTCATGGTAGCTACAAGTGGGAAAAAGGTGGTGGTCATTCCACATTCTATGGGCGTTTTGTACTTTCTGCATTTTATGAAATGGGTTGAGGCACCAGCTCCAATGGGTGGTGGGGGTGGATCAGATTGGTGTGCGAAGCATATAAAGGCAGTGATGAACATTGGTGGACCATTTTTGGGCGTTCCAAAAGCAGTTTCTGGACTTTTTTCTGTCGAAGCCAGAGATATTGCCGTTACCAGGGCATTTGCACCAGGCGTCTTGGATAAGGATGTATTTGGTCTTCAAACCTTTCAACATATGATGCGTATGACACGTACATGGGATTCAACCATGTCAATGATACCAAAAGGTGGGGAAACGATCTGGGGTGGCCTTGATTGGTCACCAGAAGGAGGCTATGACTGTGGTGGGAAAAAGGCGAAGAACAATCATAGTCGGACCACAGGCCAAAACGGGCTGGGTTCAACTAAAAGTGTGAATTATGGAAGAATTATATCATTTGGGAAGGATGCAGCTGCTGTACATTCGTCGAAATTGGAAAGGAAGGATTTTAGGGAAGCTGCCAAGGGTCATAATCATGCAGAGGCGGCATGCCATGATGTATGGACAGAGTACAATGACATGGATGTTGAGAGCTTCAAAGCTGTCTCAGATTATAAAGTTTACACTGCAGGGACGATTTTGGATCTGCTTCATTTTGTTGCTCCCAAGCTGATGGTACGTGGAGGTGCTCATTTCTCATATGGTATCGCTGACAATTTGGATGACCCGAAATACGAGCACTATAAATATTGGTCAAACCCCTTGGAAACCAAGTTACCATATGCTCCAGGCATGGAGGTTTATTCTCTGTACGGAGTTGGAGTTCCAACTGAAAGGGCGTATGTTTATAAGTTAACTAGTGGTGGGGCTGAATGTCACATCCCATTTCAAATAGACACCTCTGCAGAGGGTGGAAGTGAAGCTCCGTGTCTAAAAGGAGGGGTTTTTTCCGGTGATGGAGATGAAACTGTTCCTGCTTTAAGTGCAGGTTTCATGTGTGCAAAAGGTTGGAGAGGAAAAACCCGATTTAATCCTTCGGGGATTCCTACTTATGTAAGAGAATATGATCATGCACCTCCAGCTAATCTTCTGGAAGGCAGGGGAACACAGAGTGGTGCTCATGTTGATATAATGGGGAATTTTGCATTAATTGAGGATGTTTTAAGAGTAGCAGCAGGAGCTACAGGAGACGGGTTGGGGGGTGATCGAGTTTTCTCTGATATCTTCAAGTGGTCTAATAATATCAACTTACAACTCTAA

**>PrPDAT1-2 (675aa)**

MSNLRRRKGSEPDKFPESESLINEDEKKEEIETETEEVKKQEVVNKKKKKKKWSCIDSCCWFIGFICSVWWFLLFLYNAMPASFPQYVTEAITGPLPDPPGVKLRKEGLTAKHPVVFVPGIVTGGLELWEGHHCAEGLFRKRLWGGSFGELYKRPLCWVEHMSLDNETGLDPSGIRVRPVSGLVAADYFAPGYFVWAVLIANLARMGYEEKTMYMAAYDWRLSFQNTEVRDQSLSRIKSNIELMVATSGKKVVVIPHSMGVLYFLHFMKWVEAPAPMGGGGGSDWCAKHIKAVMNIGGPFLGVPKAVSGLFSVEARDIAVTRAFAPGVLDKDVFGLQTFQHMMRMTRTWDSTMSMIPKGGETIWGGLDWSPEGGYDCGGKKAKNNHSRTTGQNGLGSTKSVNYGRIISFGKDAAAVHSSKLERKDFREAAKGHNHAEAACHDVWTEYNDMDVESFKAVSDYKVYTAGTILDLLHFVAPKLMVRGGAHFSYGIADNLDDPKYEHYKYWSNPLETKLPYAPGMEVYSLYGVGVPTERAYVYKLTSGGAECHIPFQIDTSAEGGSEAPCLKGGVFSGDGDETVPALSAGFMCAKGWRGKTRFNPSGIPTYVREYDHAPPANLLEGRGTQSGAHVDIMGNFALIEDVLRVAAGATGDGLGGDRVFSDIFKWSNNINLQL.

**(E)**

**>*PrPDAT2* (2040bp)**

ATGGCTTCGATTCTTCGGTTTCGTAAGCTATGTTATGTAGAGCCTGTGAATTGTTCTTCATTGGGTTTGGAATCATTTCCAACCCCAAAGATTGATAAGAAAGAGGGTGTTGTTGTTTCTAATGTACTTGAAAAGCATCACGAGAAGAAGAGTAGGCAGCATAAGGAATGGAAGTGTATAGATTACTGTTGTTGGACAATTGGGTATATGTGCACTACTTGGTGGCTTCTCTTGTTTTTGTACCACTGTTTGCCTGCTACGTTGCCTGGTTTTCGGGTGCTTGAACTGCCCGGGGAGAGGCTTAAGCGAGAGGGCATAACCCCTCTTCATCCAGTTGTTTTGGTGCCAGGCATTGTAACTGGTGGGTTAGAACTTTGGGAAGGCCGGCCCTGTGCAGAGGGTCTGTTTAGGAAGCGGCTTTGGGGTGGTAGCTTTACAGAAATCTTCAAGAGGCCTTTCTGTTGGTTGGAGCATCTGGCCCTGCACAATGAGACTGGGCTCGACCCTCCAGGCATTCGGATTCGTGCTGTTCCGGGACTGGTTGCAGCTGATTATTTTGCTCCAGGATATTTTGTTTGGGCTGTTCTCATTGAGAATTTAGCGCGAATTGGTTACGAGGGGAAGAATTTGCATATGGCTGCTTATGATTGGAGACTCTCCTTCCAAAATACAGAGATACGGGACCAATCTCTTAGTAGACTGAAGAGTAAAATTGAGCTTATGTTTGTGACCAATGGCTATAAGAAAGTGGTGGTGGTGCCTCATTCCATGGGTGTAATTTATTTTCTCCACTTCCTTAAATGGGTTGAATCACCTCCCCCTATGGGAGGTGGTGGAGGTCCAGGTTGGTGTGCTAAGTACATCAAAGCAATCATGAACATCGGTCCGCCATTTCTTGGTGTTCCAAAGGCAGTTAGTAATGTATTGTCTGCGGAAGGTAAAGATGTTGCTTTTGTCAGAGCTATGGATCCAGGCGTGTTAGATTCTGAGATTCTCAGGCTTCAAACCTTAGAGCATGTGATGAGGGTATCTCGAACATGGGATTCCATCATTTCGTTGATGCCAAAAGGAGGAGAAACCATTTGGGGTAATATGGATTGGTCTCCTGAAGAAGGGTTAGATTGTGATTTGGCAAAGAAAAGATACCTAAAATCTTCTTTAAGTGACAAAAGTACCAATGATAGTGATGGAATGGTAGGTTTCTGCATAATAGATTCCATGAGATATGGAAGAATAGTTTCTTTTGGGAAGGCCACATCACAGCTGCCCTCTTCTCAGCTCCCTGCTCTTGGTTTAAAGGAACTTTTACGCTCAAGTACATCCGAGTCATCATGTGGAGAGGTTTGGACTGAATACGATGAAATGAGCAGGGATAGCATCCAGAAAGTTGCAGAAAACAAAGCTTACACGGCTAAAACTCTTCTTGATCTACTTCGCTTTGCAGCTCCAAAAATGATGCAACGAGCCGAAGCCCACTTTTCTCATGGTATAGCTGAGAATCTTGATGATCCTAAATACGCCCATTACAAGTATTGGTCCAATCCACTTGAGACCAAGTTACCTGATGCTCCAGATATGGAGATTTACAGTTTATATGGTGTTGGAATTCCAACTGAAAGGTCATATGTATACAAGCTGTCGCCTTCCGACAGGTGCAAGAGCATTCCTTACCAGATTGATAGCTCGGCCGATGGAAGTGGGAACGGTTGCTTGAAAGGTGGGGTATACTTTGTGGATGGTGACGAGAGCGTGCCTGTTTTGAGTGCGGGATTTATGTGTGCCAAAGGATGGAAGGGAAGAACTCGGTTCAATCCGTCCGGCATTGCCACGTACGTAAGGGAGTATCAGCACAAGCCACCGGCGAGTCTGTTTGAAGGGAGGGGTATAGAGAGTGGGGCTCATGTTGACATCATGGGAAATGTTGCTTTGATTGAGGATGTACTGCGAGTTGCTGCTGGTGCTTCTGGTGCGGAGATGGGAGGTGATAGGGTTTATTCGGATATCATGAGAATGTCAGAAAGAATAAATCTCAGGCTGTGA

**>PrPDAT2 (679aa)**

MASILRFRKLCYVEPVNCSSLGLESFPTPKIDKKEGVVVSNVLEKHHEKKSRQHKEWKCIDYCCWTIGYMCTTWWLLLFLYHCLPATLPGFRVLELPGERLKREGITPLHPVVLVPGIVTGGLELWEGRPCAEGLFRKRLWGGSFTEIFKRPFCWLEHLALHNETGLDPPGIRIRAVPGLVAADYFAPGYFVWAVLIENLARIGYEGKNLHMAAYDWRLSFQNTEIRDQSLSRLKSKIELMFVTNGYKKVVVVPHSMGVIYFLHFLKWVESPPPMGGGGGPGWCAKYIKAIMNIGPPFLGVPKAVSNVLSAEGKDVAFVRAMDPGVLDSEILRLQTLEHVMRVSRTWDSIISLMPKGGETIWGNMDWSPEEGLDCDLAKKRYLKSSLSDKSTNDSDGMVGFCIIDSMRYGRIVSFGKATSQLPSSQLPALGLKELLRSSTSESSCGEVWTEYDEMSRDSIQKVAENKAYTAKTLLDLLRFAAPKMMQRAEAHFSHGIAENLDDPKYAHYKYWSNPLETKLPDAPDMEIYSLYGVGIPTERSYVYKLSPSDRCKSIPYQIDSSADGSGNGCLKGGVYFVDGDESVPVLSAGFMCAKGWKGRTRFNPSGIATYVREYQHKPPASLFEGRGIESGAHVDIMGNVALIEDVLRVAAGASGAEMGGDRVYSDIMRMSERINLRL.
